# Deficiency of the paternally-expressed imprinted *Peg3* gene in mice has sexually dimorphic consequences for offspring communication and social behaviour

**DOI:** 10.3389/fnins.2024.1374781

**Published:** 2024-03-26

**Authors:** Hannah R. Tyson, David J. Harrison, Mathew J. Higgs, Anthony R. Isles, Rosalind M. John

**Affiliations:** ^1^Biomedicine Division, School of Biosciences, Cardiff University, Cardiff, United Kingdom; ^2^Behavioural Genetics Group, MRC Centre for Neuropsychiatric Genetics and Genomics, Neuroscience and Mental Health Research Institute, Cardiff University, Cardiff, United Kingdom

**Keywords:** epigenetics, imprinted genes, *Peg3*, sexual dimorphism, ultrasonic vocalisation, social behaviour

## Abstract

**Introduction:**

Imprinted genes are expressed from one parental allele as a consequence of epigenetic processes initiated in the germline. Consequently, their ability to influence phenotype depends on their parent-of-origin. Recent research suggests that the sex of the individual expressing the imprinted gene is also important. We have previously reported that genetically wildtype (WT) dams carrying and caring for pups mutant for *PEG3* exhibit anxiety-like behaviours and their mutant pups show a reduction in ultrasonic vocalisation when separated from their mothers. Sex-specificity was not examined.

**Methods:**

WT female mice were mated with WT, heterozygous *Peg3*^−/+^ or homozygous *Peg3*^−/−^ studs to generate all WT (control), 50:50 mixed or 100% mutant litters, respectively, followed by behavioural assessment of both dams and their pups.

**Results:**

We reproduced our original finding that WT dams carrying and caring for 100% mutant litters exhibit postpartum anxiety-like symptoms and delayed pup retrieval. Additionally, these WT dams were found to allocate less time to pup-directed care behaviours relative to controls. Male *Peg3*-deficient pups demonstrated significantly reduced vocalisation with a more subtle communication deficit in females. Postweaning, male mutants exhibited deficits across a number of key social behaviours as did WT males sharing their environment with mutants. Only modest variations in social behaviour were detected in experimental females.

**Discussion:**

We have experimentally demonstrated that *Peg3* deficiency confined to the offspring causes anxiety in mouse mothers and atypical behaviour including deficits in communication in their male offspring. A male-specific reduction in expression *PEG3* in the fetally-derived placenta has previously been associated with maternal depression in human pregnancy. Maternal mood disorders such as depression and anxiety are associated with delays in language development and neuroatypical behaviour more common in sons. *Peg3* deficiency could drive the association of maternal and offspring behavioural disorders reported in humans.

## Introduction

Genomic imprinting is an epigenetic process initiated in the mammalian germline which results in the monoallelic expression of specific genes in offspring (Surani, 1998). Imprinted genes function in a myriad of processes vitally important for the reproductive success of mammals including foetal growth, placental development, adult metabolism, cognition and social behaviours (Tucci et al., 2019). Their functions are highly dosage-sensitive and relatively subtle increases in expression can have a marked effect on phenotype (Andrews et al., 2007; da Rocha et al., 2009; Tunster et al., 2010). Imprinted genes can also respond epigenetically *in utero* to dietary interventions leading to persistent changes in their expression (Van De Pette et al., 2017, 2022). The potential for imprinted genes to influence phenotype in a sexually dimorphic manner remains a relatively underexplored area of research.

*Paternally expressed gene 3* (*Peg3*, also known as *Pw1*) is a paternally expressed, maternally DNA methylated imprinted gene that encodes a Krüppel-type zinc finger protein (Kuroiwa et al., 1996). In mice, *Peg3* is widely expressed during development in mesodermal, endodermal and neural tissues, with expression persisting into adulthood primarily in the brain (Li et al., 1999) and in specific adult stem cell populations (Besson et al., 2011). In the placenta *Peg3* is expressed in a number of cell types with endocrine capacity (Tunster et al., 2018). *Peg3* expression is predominantly monoallelically expressed, but biallelic expression has been reported in the choroid plexus and subregions within the mouse hypothalamus (Perera et al., 2015). Targeted deletion of the paternal *Peg3* allele in mice results in phenotypes related to foetal growth, placental development, metabolism and behaviour. Briefly, when compared to their WT littermates *Peg3*-deficient foetuses are lighter and born low birth weight, retaining this weight deficit into adulthood (Li et al., 1999; Kim et al., 2013; Denizot et al., 2016; Tanaka et al., 2022). Deficiency in *Peg3* has a sexually dimorphic consequence for placental development. Male *Peg3*-deficient placenta exhibit a significant reduction of two placental lineages (Tunster et al., 2018): the spongiotrophoblast which is a major endocrine cell type (Tunster et al., 2016) and the glycogen cells thought to be important for foetal growth and parturition (Coan et al., 2006). Female *Peg3*-deficient placenta are substantially less impacted by loss of *Peg3* function (Tunster et al., 2018). *Peg3*-deficient pups emit fewer ultrasonic vocalisations (USVs) when separated (McNamara et al., 2018). USVs are a form of communication essential for invoking maternal care behaviours such as pup retrieval, nursing, and nest building (D'Amato et al., 2005). *Peg3*-deficiency decreases sucking by pups (Curley et al., 2004; Kim et al., 2013). Sucking is another important stimulus for maternal behaviour, in part, through the activation maternal oxytocin neurons (Caldwell et al., 2017). As adults, *Peg3*-deficient animals exhibit sex-specific alterations in metabolism. Male mutant mice accumulate more body fat, and to have reduced lean mass and reduced thermogenesis compared to their WT littermates (Curley et al., 2005), a phenotype not apparent in female mutants (Tanaka et al., 2022). Both male and female *Peg3*-deficient mice exhibit have abnormal behaviour related to reproduction. Mutant dams exhibit deficits in pup retrieval and nest building with increased litter loss, and possess a reduced number of oxytocin-expressing neurons (Li et al., 1999; Curley et al., 2004). In addition to direct consequences of loss-of-function, we reported that genetically unmodified dams with *Peg3* mutant offspring also show deficits in maternal behaviour (McNamara et al., 2018). Specifically, WT dams carrying and caring for litters consisting entirely of *Peg3*-deficient pups exhibit enhanced maternal novelty reactivity during pregnancy and, postpartum, delayed sniffing and retrieval of their pups, alongside anxiety-like behaviours. Unlike WT males, *Peg3*-deficient male mice do not improve their sexual performance with experience (Swaney et al., 2008). Male mutants also exhibit reduced aggressive behaviour and reduced social dominance (Tanaka et al., 2022). Behavioural differences in mature male and female mice may be attributed to differences in level of hormones relating to reproduction, such as testosterone and estrogens (Tanaka et al., 2022) but may reflect a sexually dimorphic role for this gene as observed in the placenta (Tunster et al., 2018).

The importance of studying the sex-specific functions for *Peg3* comes from studies on human *PEG3*. Lower expression of placental *PEG3* has been reported to occur in pregnancies where mothers are clinically diagnosed with depression or report symptoms of depressions via questionnaires (Janssen et al., 2016a). However, this association was only apparent when the foetus was male. Whilst postnatal depression is a well-known complication of pregnancy, depression during pregnancy is more common, with many mothers experiencing clinically significant symptoms of both depression and anxiety whilst pregnant (Janssen et al., 2016b). Infants of mothers with mood disorders are more likely to be born preterm, and to be low birth weight with increased risk of mortality and morbidity (Henrichs et al., 2010; Liu et al., 2012). Maternal depression and anxiety symptoms have been linked to language delays and emerging emotional difficulties in male infants (Savory et al., 2020) and, more specifically, prenatal anxiety (Zhang et al., 2023). As exposed children get older, other sexually dimorphic behavioural features have been linked to maternal depression and/or anxiety with deficits in attention, cognitive problems and externalising behaviour observed more commonly in boys (Van den Bergh et al., 2005; Talge et al., 2007; Raikkonen et al., 2015; Lahti et al., 2017) and anxiety, depression and internalising behaviour in girls (Glover and Hill, 2012). Whilst the expression level of *PEG3* has not been explicitly linked to aspects of human infant behaviour, DNA methylation of the *PEG3* differentially methylated region in umbilical cord blood leukocytes positively associates with externalising and negative affectivity, although not stratified by sex (Fuemmeler et al., 2016).

Based on clinical and preclinical findings linking *PEG3* to prenatal maternal mood, and the link between maternal depression and language delays in male human infants, the aim of this study was to explore early communication and social behaviours in *Peg3*-deficient male and female mouse offspring. We compared dams and individuals from litters composed entirely of WT animals with litters where the dam was WT and all the pups were *Peg3*-deficient, as previously (McNamara et al., 2018). We included a new group composed of ~50% *Peg3*-deficient and ~50% WT pups. The inclusion of a mixed genotype litter allowed for the comparison of maternal care differentially directed towards the WT or mutant offspring, as has been observed with the imprinted *Magel2* gene where dams preferentially retrieved WT over *Magel2*-deficient pups (Bosque Ortiz et al., 2022). Additionally, this study design allowed us to ask whether there were behavioural alterations in WT littermates sharing their perinatal environment with mutant pups as has been reported for mice lacking *Neuroligin-3* or overexpressing *Phlda2* (Kalbassi et al., 2017; Harrison et al., 2021).

## Materials and methods

### Experimental design

Mice were housed in a regulated holding room [temperature (21°C ± 2)] and humidity (50% ± 10%) maintained on a 12-h light–dark cycle with lights coming on at 06:00 h. All cages contained a cardboard tube, chew-stick and nestlet bedding, and had free access to enriched chow (Formulab Diet 5008, TestDiet, United Kingdom) and tap water. Cages were cleaned out weekly at a regular time to ensure minimal disruption to behavioural testing and animal breeding. The *Peg3KO* colony (Li et al., 1999) was maintained on the 129S2/SvHsd (129) strain background and bred alongside an in-house 129 colony. WT 129 females aged between 8 and 14 weeks were used to generate control and experimental litters. Successful mating was determined by the presence of a vaginal plug and recorded as gestational day (G) 1. Where possible, the plugged females were housed with other female mice plugged by a stud of the same genotype. At G17, pregnant females were singly housed to ensure the biological dam of each litter could be determined. *Peg3KO* genotyping of offspring was performed using primers CGTTGGCTACCCGTGATATT and TATGCACACAGCCTCTGCTC as previously described (McNamara et al., 2018).

**Figure 1:**
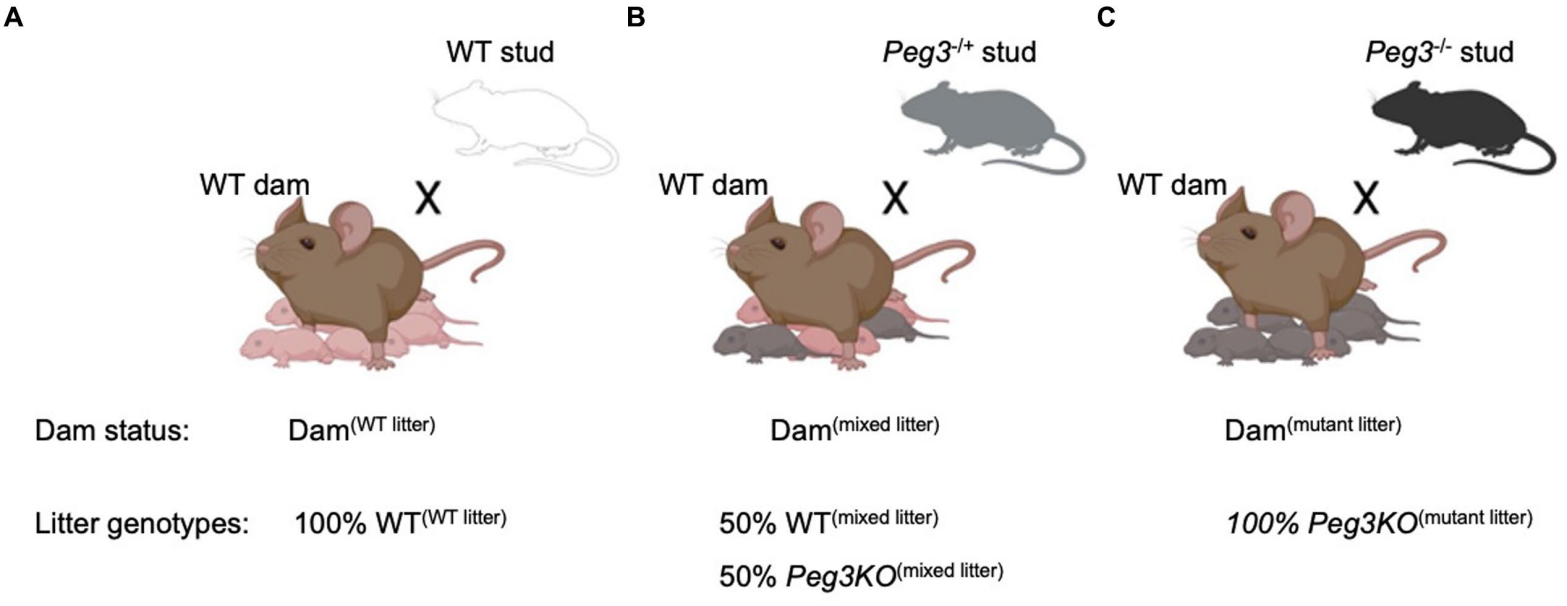
Schematic of the breeding design used to generate experimental dams and offspring **(A)** Control group = 100% wild-type litter. Wild-type (WT) females mated with WT male to produce litters composed entirely of WT pups WT^(WT Litter)^. **(B)** Mixed genotype litter: WT females mated with heterozygous *Peg3*^−/+^ male to produce litters composing ~50% *Peg3*^+/+^ [WT^(Mixed Litter)^] pups and ~50% heterozygous mutant Peg3^+/−^ [*Peg3KO*^(Mixed Litter)^ pups]. **(C)** 100% mutant litter: WT females mated with homozygous *Peg3*^−/−^ male to produce litters composed entirely of mutant pups [*Peg3KO*^(Mutant Litter)^]. Data used from litter sizes > 4 and, for mixed genotype litters, where the proportion of transgenic pups within the litter was between 45% and 60%.

Three breeding strategies were used to generate the experimental cohorts: (A) WT females mated with WT studs to generate all WT litters as controls with dams referred to as Dam^(WT litter)^ and offspring referred to as WT^(WT litter)^; (B) WT females mated with heterozygous *Peg3*^+/−^ (KO) studs to generate mixed litters of WT and *Peg3^−/+^* (KO) pups with dams referred to as Dam^(mixed litter)^ and offspring referred to as WT^(mixed litter)^ or *Peg3KO*^(mixed litter)^; and (C) WT females mated with homozygous *Peg3*^−/−^ (KO) studs to generate 100% *Peg3KO* litters with dams referred to as Dam^(mutant litter)^ and offspring referred to as *Peg3KO*^(mutant litter)^ (Figure 1). All three groups were housed in the same room, bred and studied concurrently. Litter sizes from Dam^(mutant litter)^ were smaller at P2 [χ^2^(2) = 10.28, *p* = 0.006] compared to Dam^(WT litter)^ (*P*= 0.01) and Dam^(mixed litter)^ (*p*= 0.04; Supplementary Figure 1). Consequently, litter size was included as a covariate in all maternal behaviour analyses.

### Behavioural testing of dams

Day of birth was designated as postnatal day (P) 0. Dams and their litters remained undisturbed until the first day of testing (P2). At weaning (P21), offspring to be used for further behavioural testing were housed in same-sex cages of four where possible, preferentially keeping mice from the same birth litter together. To maintain consistency between the pre-and postnatal environment, offspring from the mixed litters were, where possible, housed into cages containing two WT^(mixed litter)^ pups and two *Peg3KO*^(mixed litter)^ littermates.

### Ultrasonic vocalisations (USVs)

USVs were recorded using an ultrasonic microphone (Avisoft UltraSoundGate 116Hb, Avisoft Bioacoustics, Berlin, Germany) and accompanying software (Avisoft Bioacoustics USGH). As in previous research, recording settings included a sampling rate of 250 kHz, format 16 bit, and a high pass filter set to 15 kHz in order to reduce background noise (McNamara et al., 2018). Raven Pro 1.6 software (Bioacoustics Research Program, Cornell Lab of Ornithology, Ithaca, NY) was used to generate spectrograms with a 256-sample Hann window, one frame per second. Raven’s built in call detection was then used to detect calls, with a selection of spectrograms manually checked to determine accurate detection.

### Pup retrieval and maternal USVs

At P3 and P9 dams underwent a pup retrieval test with USVs from both dam and pups recorded immediately prior to the trial starting. To begin the trial, dams were placed into a sound-attenuating chamber, where USVs were recorded for 180 s. Following this, the dam was transferred to an empty cage outside of the testing room whilst the litter USVs were recorded (see below). After recording, four pups were spaced equidistantly within the home cage and the dam reintroduced, indicating time zero. Time taken to sniff and retrieve each pup was recorded. A successful retrieval was defined as the pup being placed well within the nest. The trial ended when all pups had been successfully retrieved and the dam was crouched over the nest, or after 900 s. Any pups that had not been successfully retrieved were given the maximum score of 900 s. Average retrieval times were analysed in a 2-way ANCOVA. During the test the dams’ behaviour was recorded, and subsequently analysed in two categories: pup-directed and non-pup directed behaviour (categories provided in Supplementary Table 1) using litter size as a covariate. Category scores were calculated by summing the time spent engaged in each behaviour making up the category.

### Elevated zero maze (ELM) and light dark box (LDB)

Anxiety-like behaviour was measured using the EZM and LDB tasks, carried out at P4 and P7, respectively. At the beginning of each trial, the dam was placed in the enclosed, dark section of the apparatus and allowed to freely explore for 300 s. Time spent in, latency to enter, and number of crosses into the anxiogenic open/light section were analysed, in addition to the number of stretch-attend postures displayed.

### Pup ultrasonic vocalisation

Prior to performing the pup retrieval task on P3 and P9, four pups (two males and two females determined by anogenital distance) were placed equidistant at the opposite end of the cage to the nest, directly under the ultrasonic microphone. USVs from all four pups were simultaneously recorded for 180 s. In addition, USVs were recorded for individual pups at P2, P4, P6, P8, and P10 for each litter in a sound attenuating chamber, and call number was analysed. Body temperature of the pups was measured using an infrared thermometer on the back of the pups neck, pre, and post USV measurement. The difference in body temperature was recorded and used as a covariate in the USV analyses.

### Direct social interaction

DSI was used to measure the tendency of mice to interact with an unfamiliar female mouse of a different strain (CD1). Testing took place at P28 before the subjects had reached sexual maturity. The test was recorded and the subject mouse behaviour analysed by grouping into ‘Social’ or ‘Non-social’ (Supplementary Table 2) as in previously described (Harrison et al., 2020, 2021).

### Social propinquity test

The social propinquity test was used to measure anxiety-like and social behaviours by exploring the tendency for mice to maintain close physical proximity with an unfamiliar mouse of the same sex (increased sociability) and the tendency to avoid an aversive arena (anxiety-like behaviour; Tuttle et al., 2017; Harrison et al., 2020, 2021). Testing took place at P35. For each 60-min trial, two genotype-matched, non-cage mate and unrelated mice were placed into a clear Perspex cube (30 cm x 30 cm), illuminated brightly from below to create an aversive arena. Each cube contained one cardboard tube providing a single, sheltered space. Using video recordings, latency to share the tube, proportion of shared tube time and vacant tube time were measured, with greater time spent sharing the tube used to indicate increased sociability (Harrison et al., 2020).

### Three chamber test and scent marking

The three-chamber test is a commonly used method of assessing sociability in mice (Moy et al., 2004) consisting of three identically sized interconnecting chambers. Testing took place at P63. Each end-chamber of the apparatus contained a wire mesh cage (6 cm × 10 cm), one empty and one containing an unfamiliar host mouse of a different strain (CD1) and opposite sex to the test mouse. Whatman chromatography paper (3 mm, GE Healthcare Whatman™, Fisher-Scientific) cut to 14 × 29 cm was used to line the floor of each chamber for scent marking analysis and to improve contrast between the mouse and the chamber for later automated video analysis (Ethovision XT, Noldus, United Kingdom). At the beginning of each trial, test mice were placed in the middle chamber perpendicular to the chamber entrances and allowed to freely explore for 180 s. Parameters analysed included the total length of time spent in each chamber, number of entries and latency to enter each chamber. On completion of the trial, the scent-marked paper was removed and allowed to dry. Once dry, the outline of any scent marking was traced with a pen under an ultraviolet light (Bio-rad industries). Area marked was then calculated by counting the total number of squares of a 1 cm^2^ grid overlay containing a scent mark. Using the work of Rienecker et al. (2020) as a guide, marks covering more than 4 consecutive squares were not included, as this was indicative of urine rather than scent marking. A higher score was indicative of greater scent marking and taken to represent increased territorial behaviour or social dominance.

### Courtship USVs

Courtship behaviours and USVs were measured using the two-phase male–female social interaction test (Yang et al., 2013). Testing took place at P70. Briefly, to begin the trial, the male test mouse was placed into a clean cage and baseline USVs were recorded for 60s. For the initial phase, a female mouse (confirmed to be in oestrous via vaginal swab and cytology) was introduced to the cage at the opposite end to the male. USVs and social interactions were recorded for 300 s with the experimenter outside of the testing room. Following the initial phase, the female mouse was removed and placed into a clean cage outside of the testing room whilst USVs from the male were recorded in isolation for a further 3 min. The reunion phase began when the female mouse was reintroduced to the testing cage and USVs and interactions were both recorded for a further 180 s.

## Statistics

All data were analysed using IBM SPSS software (version 27). Data are presented as mean and standard error of the mean (SEM) unless otherwise stated. Sample size required for each group was calculated using G*Power (Faul et al., 2007). A standardised effect size (d) of 0.7 was used as recommended (Wahlsten and Bulman-Fleming, 1987) to detect small-moderate effects in animal research. A sample size of 10 dams per group was predicted to be sufficient for power (1 − β) = 0.09 with a type 1 error rate (α = 0.05) in one-way Analyses of Variance (ANOVAs) and Analyses of Covariance (ANCOVAs). Differences in litter size between experimental groups were assessed using a Kruskal-Wallis H test due to violations of normality in both Dam^(mixed litter)^ and Dam^(mutant litter)^ groups. As a significant difference in litter size was observed between Dam^(mutant litter)^ and Dam^(WT litter)^, this was subsequently included as a covariate in all analyses examining group differences in aspects of maternal care and maternal anxiety-like behaviours. Between-group differences in maternal behaviour for individual tests and the unified maternal anxiety score were assessed using one-way Analysis of Covariance tests (ANCOVA) with litter size used as the covariant. Where a significant result was found, *post-hoc* pair-wise comparisons were carried out with Bonferroni corrections to determine which groups significantly differed from the others.

## Results

### Catch-up growth of *Peg3KO* mice raised in all mutant litters but not mixed litters

A two-way mixed ANCOVA was run to explore the effect of genotype on pre-weaning weight at P2-P10 in both male and female pups, with litter size as a covariate. Greenhouse–Geisser correction values are reported due to a violation of Mauchly’s assumption of Sphericity. In males, a significant time*genotype interaction was observed [Time*Genotype: *F*(7.36, 238.06) = 8.80, *p* < 0.001]. A significant main effect of genotype on male pup weight at P2, P4, P8 and P10 was observed, but not at P6 [Genotype P2: *F*(3,100) = 7.97, *p* < 0.001], [Genotype P4: *F*(3,100) = 3.59, *p* < 0.016], [Genotype P8: *F*(3,99) = 9.26, *p* < 0.001], and [Genotype P10: *F*(3,99) = 10.91, *p* < 0.001]. *Post-hoc* tests demonstrated that both *Peg3KO*^(mixed litter)^ and *Peg3KO*^(mutant litter)^ males weighed less than WT^(WT litter)^ male pups at P2 (*p < 0*.001). At P4, *Peg3KO*^(mixed litter)^ male pups weighed less than WT^(mixed litter)^ male pups from the same litter (*p* = 0.03). At both P8 and P10, *Peg3KO*^(mixed litter)^ male pups consistently weighed less than males in all other groups (*p < 0*.001 across each group at both P8 and P10; Supplementary Figure 2A).

In females, a similar pattern of results were observed (Supplementary Figure 2B), with a significant time*genotype interaction [Time*Genotype: *F*(6.95, 227.14) = 10.67, *p* < 0.001]. A significant main effect of genotype was observed for female pup weight at all time points with the exception of P6, [Genotype P2: *F*(3,100) = 23.30, *p* < 0.001], [Genotype P4: *F*(3,100) = 15.96, *p* < 0.001], [Genotype P6: *F*(3,100) = 1.76, *p* = 0.16], [Genotype P8: *F*(3,99) = 14.87, *p* < 0.001], and [Genotype P10: *F*(3,99) = 15.46, *p* < 0.001]. At both P2 and P4, both *Peg3KO*^(mixed litter)^ and *Peg3KO*^(mutant litter)^ females weighed significantly less than either WT^(WT litter)^ or WT^(mixed litter)^ females (*p < 0.*001 for each comparison). At P8 and P10, *Peg3KO*^(mixed litter)^ female pups weighed significantly less than females from every other group (*p* ≤ 0.001 across all comparisons).

Post weaning, *Peg3KO*^(mixed litter)^ male pups maintained a weight deficit up to 14 weeks old whilst *Peg3KO*^(mutant litter)^ male weight had caught up to the WT^(WT litter)^ male weight by 4 weeks of age (Supplementary Figure 3A). *Peg3KO*^(mixed litter)^ female pups similarly remained lighter than females in other groups. In addition, by 14 weeks WT^(mixed litter)^ females weighed significantly less than WT^(WT litter)^ females (Supplementary Figure 3B).

In summary, all *Peg3*-deficient pups weighed less than WT pups 2 days postpartum. At later timepoints *Peg3KO*^(mutant litter)^ pups demonstrated catch up growth. In contrast, *Peg3KO*^(mixed litter)^ pups, with the same *Peg3* genotype but sharing their environment with WT pups, did not recover to WT weights, suggestive of intra-litter competition rather than intrinsic growth restriction.

### Maternal anxiety-like behaviours in WT mothers carrying and caring for *Peg3KO* pups

**Figure 2:**
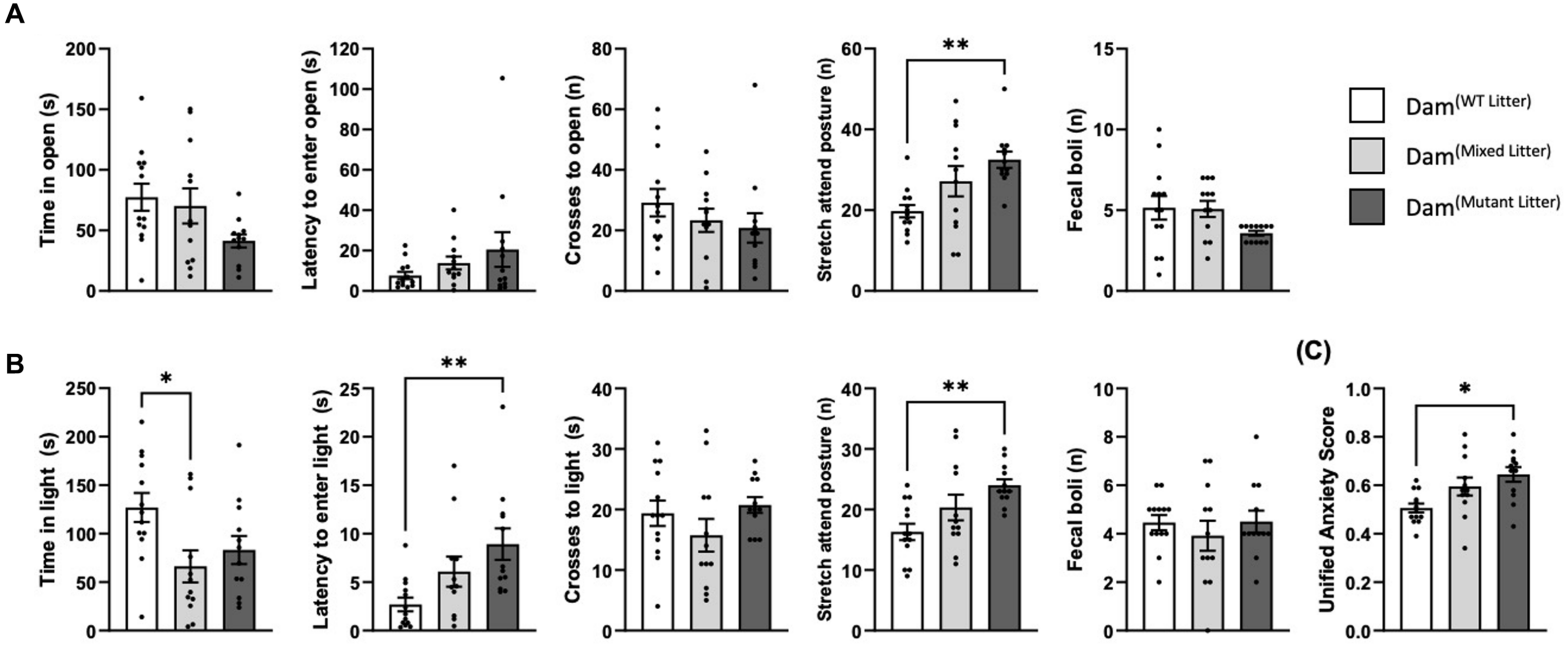
Maternal anxiety-like behaviour. Analysed using ANCOVA controlling for litter size as a covariate. **(A)** Elevated zero maze, 4 days post-partum. Dam^(Mutant Litter)^ demonstrated significantly more stretch attend postures than Dam^(WT Litter)^ (*p* = 0.01). No further significant differences were detected. **(B)** Light Dark Box, 7 days post-partum. Dam^(Mutant Litter)^ demonstrated significantly more stretch attend postures than Dam^(WT Litter)^ (*p* = 0.01). Differences were observed for both time spent in, and time taken to enter the anxiogenic section of the LDB. Dam^(Mixed Litter)^ spent significantly less time in the light than Dam^(WT Litter)^ (*p* = 0.04) and Dam^(Mutant Litter)^ were slower to enter the anxiogenic section than Dam^(WT Litter)^ (*p* = 0.01). No further significant differences were detected. **(C)** Unified Anxiety Score. When all anxiety-like outcome measures were combined to generate a unified anxiety score, Dam^(Mutant Litter)^ demonstrated greater anxious-like behaviour than Dam^(WT Litter)^ (*p* = 0.02). Number of dams: Dam^(WT Litter)^ = 13, Dam^(Mixed Litter)^ = 12, and Dam^(Mutant Litter)^ = 12. Data are mean ±SEM. *p ≤ 05, **p ≤ 0.01.

Two different tests were used to probe anxiety-associated behaviours: the elevated zero maze (EZM) and the Light Dark Box (LDB). In both tests, Dam^(mutant litter)^ demonstrated a greater number of stretch-attend postures than Dam^(WT litter)^ [EZM: *F*(2,34) = 5.18, *p* = 0.01; Figure 2A and LDB: *F*(2,34) = 4.87, *p* = 0.01, *p* = 0.01; Figure 2B]. Dam^(mixed litter)^ took longer to enter the light and spent less time in the light than Dam^(WT litter)^ in the LDB [*F*(2,24) = 4.92, *p* = 0.01, *p* = 0.01; and *F*(2,34) = 4.87 *p* = 0.01 *p* = 0.04 respectively; Figure 2B].

Anxiety-related outcome measures were combined to generate unified anxiety scores for each model as described (Supplementary Table 3). A one-way ANCOVA controlling for litter size indicated that litter genotype had a significant effect on maternal anxiety [*F*(2,34) = 4.52, *p = 0*.02; Figure 2C]. This was driven by Dam^(mutant litter)^ yielding a greater anxiety score than Dam^(WT litter)^ (*p* = 0.02). When taken together, this data show that Dam^(mutant litter)^ demonstrate the greatest anxiety-related behaviour compared to the other groups, validating our previous finding (McNamara et al., 2018). Whilst Dam^(mixed litter)^ scores were intermediate between that of Dam^(WT litter)^ and Dam^(mutant litter)^, they were not significantly different to Dam^(WT litter)^.

### Maternal caregiving behaviour altered in WT dams carrying and caring for *Peg3KO* pups

**Figure 3:**
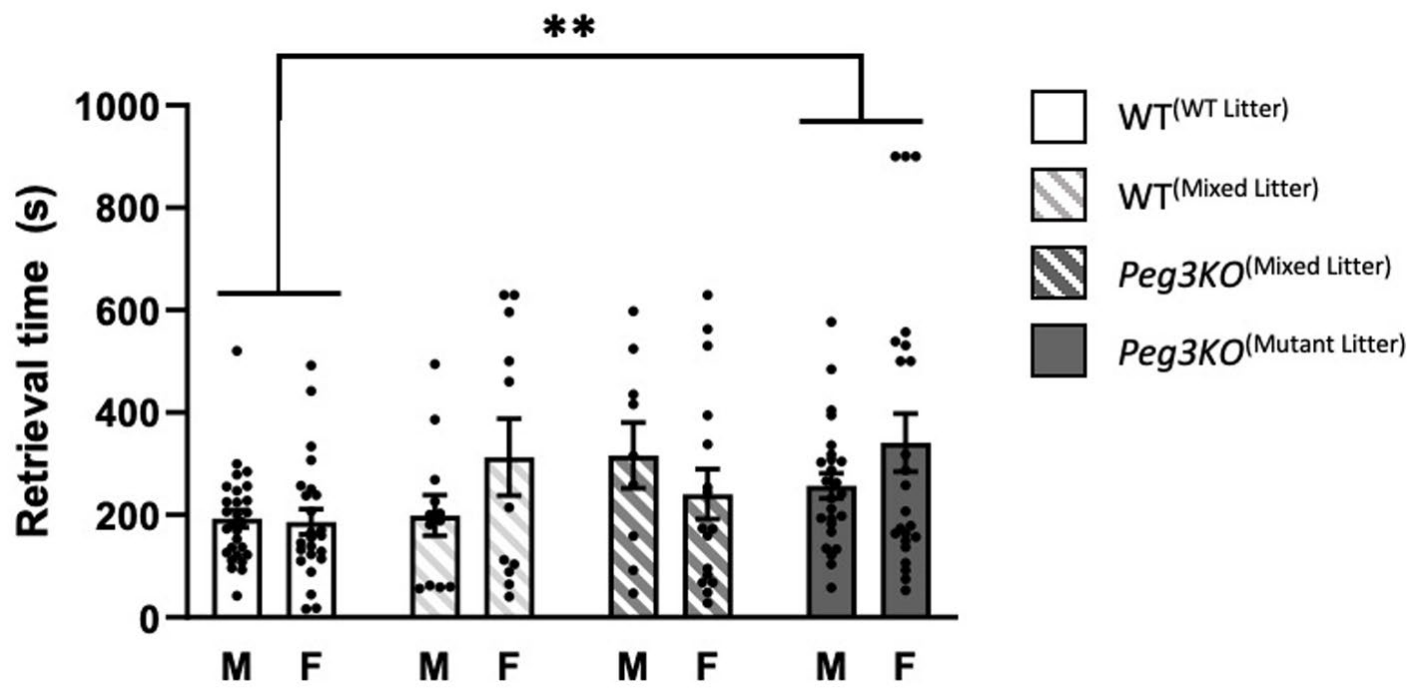
Pup retrieval at P3. Analysed using ANCOVA controlling for litter size as a covariate. Average time taken to retrieve each pup by genotype and sex. A significant main effect of genotype was detected, with *Peg3KO*^(Mutant Litter)^ being retrieved slower than WT^(WT Litter)^ at P3. Number of offspring: WT^(WT Litter)^ M = 16, F = 20; WT^(Mixed Litter)^ M = 16, F = 15; *Peg3KO*^(Mixed Litter)^ M = 13, F = 13, and *Peg3KO*^(Mutant Litter)^ M = 14, F = 14. Data are mean ±SEM. **p ≤ 0.01.

Previous research has shown that a disruption of gene function or the sex of the pup may alter maternal preference and response time in the pup retrieval task (Moore and Morelli, 1979; Deviterne and Desor, 1990; Bowers et al., 2013). Previously we reported delayed retrieval on P2 of all mutant litters by WT females. Here, the time taken to sniff and retrieve four pups at P3 and P9 was recorded and a two-way ANCOVA was run to determine whether dams could discriminate between the genotype and/or sex of their pups. This revealed a significant main effect of genotype on the average time for pups to be retrieved [*F*(3,140) = 3.55, *p* = 0.01; Figure 3]. *Peg3KO*^(mutant litter)^ pups were retrieved on average 109 s slower than WT^(WT litter)^ pups at P3 (*p* = 0.01), demonstrating that Dam^(mutant litter)^ take longer to retrieve their pups than Dam^(WT litter)^, consistent with the previous study (McNamara et al., 2018). However, no significant interaction between genotype and sex [*F*(3,140) = 1.73, *p* = 0.17], or main effect of sex [*F*(1,140) = 0.88, *p* = 0.35] were detectable. At P9, no significant differences were detectable (Supplementary Figure 4).

**Figure 4:**
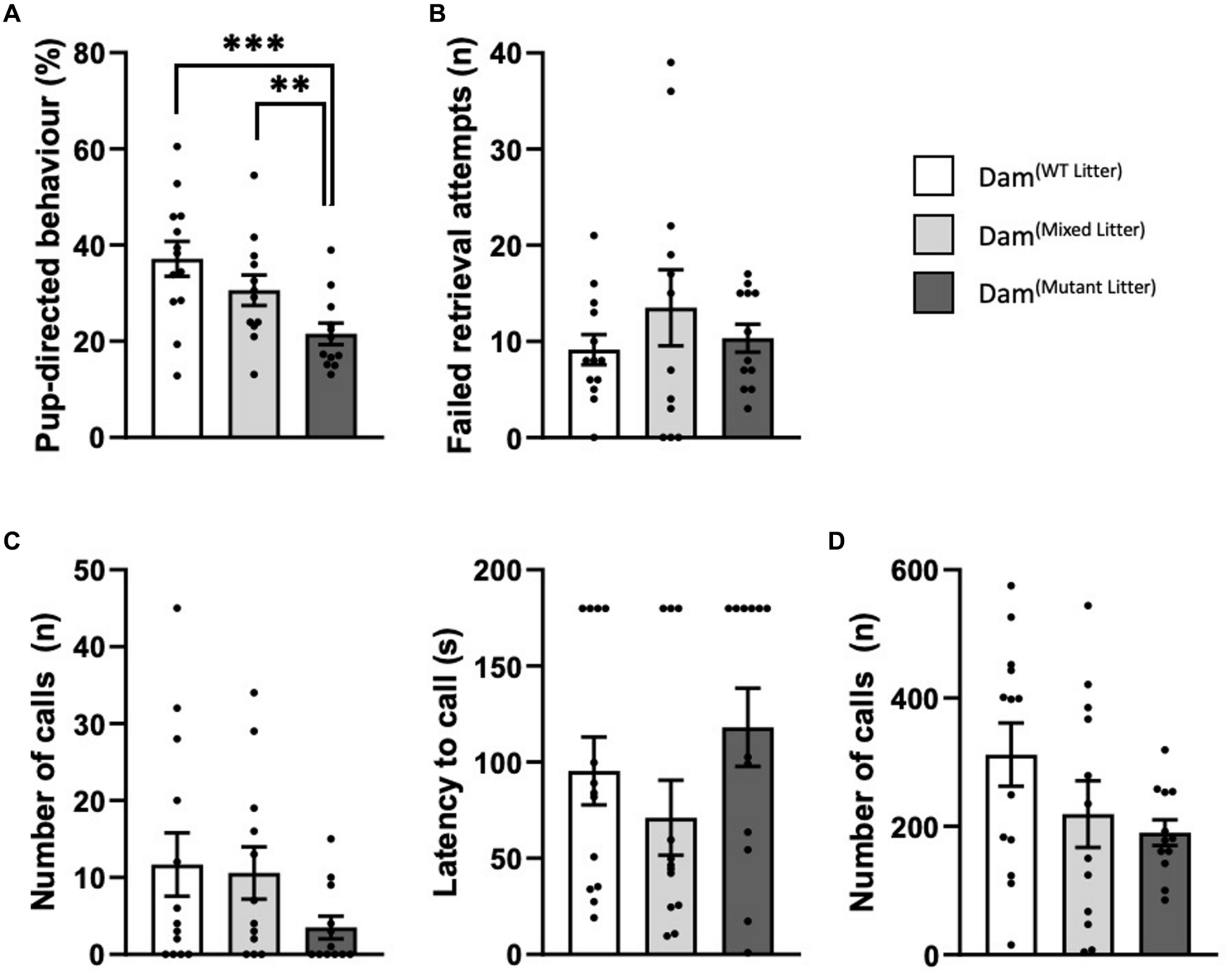
Maternal behaviour during the pup retrieval task at P3. Analysed using ANCOVA controlling for litter size as a covariate. **(A)** Percentage time engaged in pup-directed behaviour. Dam^(Mutant Litter)^ spent a significantly smaller percentage of time engaged in pup-directed behaviour than both Dam^(WT Litter)^ and Dam^(Mixed Litter)^, **(B)** Average number of failed retrieval attempts. **(C)** Number and latency to call for dam. **(D)** Combined number of calls from 4 pups. Number of dams: Dam^(WT Litter)^ = 13, Dam^(Mixed Litter)^ = 12 and Dam^(Mutant Litter)^ = 12. Data are mean ±SEM. **p ≤ 0.01, ***p < 0.001.

During the pup retrieval task, mean percentage of time dams were engaged in pup-directed and non-pup directed behaviours were measured (Figure 4; Supplementary Table 4). There was a statistically significant difference in the percentage of time engaged in pup-directed activity at P3 [*F*(2,34) = 8.10, *p* < 0.001; Figure 4A]. Dams of 100% mutant litters spent considerably less time engaged in pup-directed behaviour at P3 compared to Dam^(WT litter)^ (*p* ≤ 0.001). Dam^(mixed litter)^ showed an intermediate phenotype with significant differences compared to Dam^(mutant litter)^ (*p* ≤ 0.01). Despite the significant differences in the average time taken to retrieve pups and in time engaged in pup-directed behaviour, there were no statistically significant differences between the dams in the number of failed retrieval attempts before pup was successfully returned to nest at P3 [*F*(2,34) = 0.69, *p* = 0.51; Figure 4B]. At P9 we were unable to detect differences in these behaviours (Supplementary Figure 5; Supplementary Table 5).

USVs were recorded for 3 min from the isolated dam and the group of four pups immediately prior to reintroducing the dam to the home cage. At P3 there were no significant differences in the number of dam-emitted USVs [Group: *F*(2,34) = 0.86, *p* = 0.43] or latency to call [*F*(2,34) = 0.96, *p* = 0.40; Figure 4C]. The number of calls from 4 pups of *Peg3KO*^(mutant litter)^ [*F*(2,34) = 2.02, *p* = 0.15; Figure 4D] was not significantly different compared to WT^(WT litter)^. At P9 there were no detectable differences (Supplementary Figure 5).

### Isolation-induced USVs

**Figure 5:**
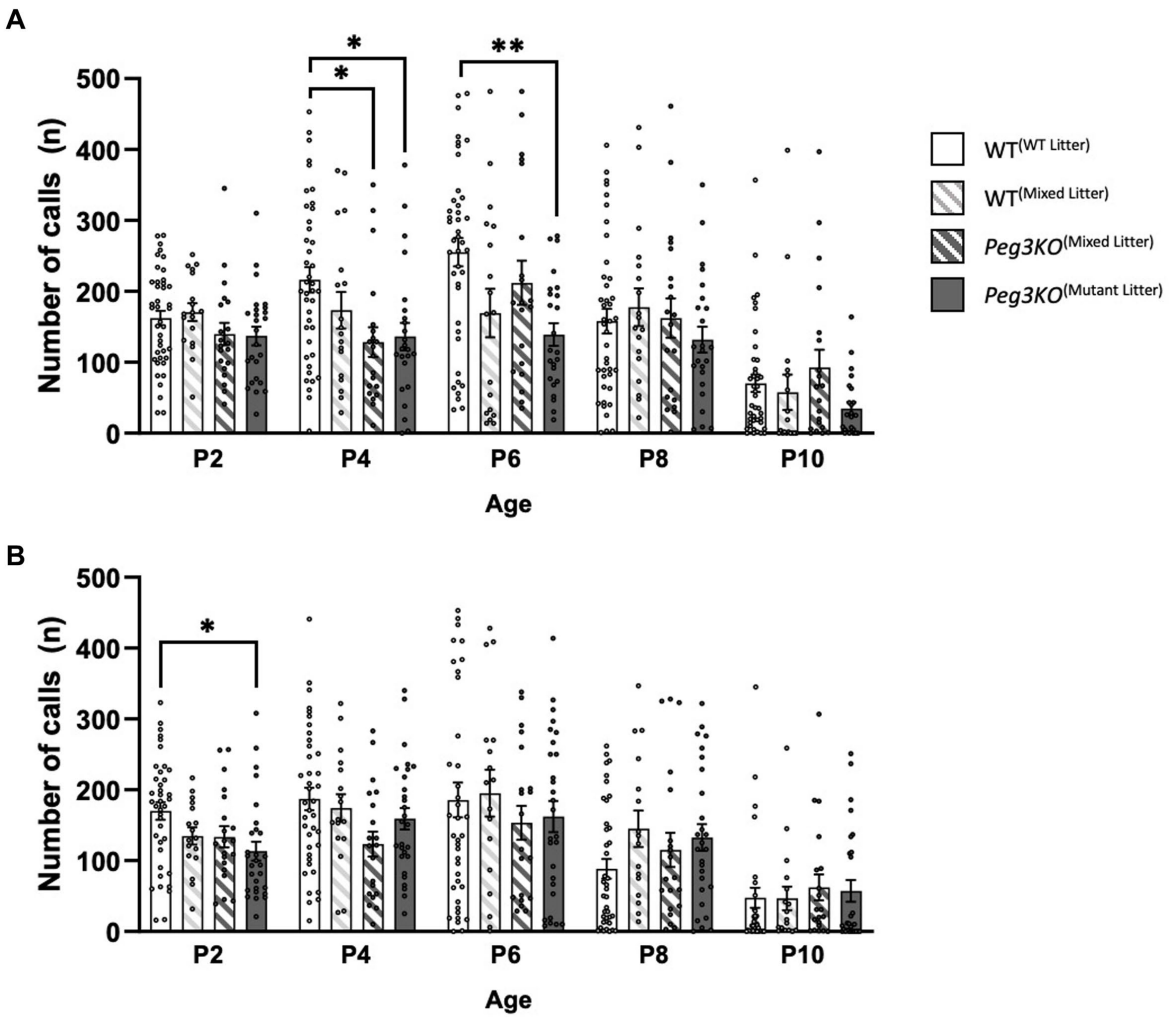
Neonatal isolation induced USVs of individual male and female pups aged P2 to P10. Analysed using ANCOVA controlling for body temperature as a covariate. **(A)** Number of calls at each age for male pups. **(B)** Number of calls at each age for female pups. Males: WT^(WT Litter)^ = 41, WT^(Mixed Litter)^ = 18, *Peg3KO*^(Mixed Litter)^ = 20, *Peg3KO*^(Mutant Litter)^ = 25; Females: WT^(WT Litter)^ = 39, WT^(Mixed Litter)^ = 17, *Peg3KO*^(Mixed Litter)^ = 20, *Peg3KO*^(Mutant Litter)^ = 28. Data are mean ±SEM. ^*^p < 0.05, ^**^p < 0.01, ^***^p < 0.001.

Individual isolation-induced USVs were recorded at P2, P4, P6, P8 and P10 in a sound attenuating chamber. Two-way mixed ANCOVAs, with Greenhouse–Geisser correction values, demonstrated that in both males and females, there was a significant interaction effect between timepoint and genotype on the number of calls: Males: [Timepoint*Genotype: *F*(11.09, 365.99) = 2.23, *p* = 0.023] and Females: [Timepoint*Genotype: *F*(3,100) = 3.81, *p* = 0.012]. In males, there was a significant effect of genotype at P4 and P6 (Figure 5A). The effect at P4 and P6 remained significant after correcting for multiple comparisons [*F*(3,99) = 4.51, *p* = 0.005 and *F*(3,99) = 5.01, *p* = 0.003, respectively]. Mutant males from *Peg3KO*^(mutant litter)^ and *Peg3KO*^(mixed litter)^ called significantly fewer times than WT^(WT litter)^ males, with *Peg3KO*^(mutant litter)^ males making 80 (*p* = 0.019) fewer calls and *Peg3KO*^(mixed litter)^ making 88 (*p* = 0.014) fewer calls on average compared to WT^(WT litter)^ males at P4. At P6, a significant difference was found between WT^(WT litter)^ males and *Peg3KO*^(mutant litter)^ males with the latter calling on average 116 times fewer in comparison across trials. In females, a significant effect of genotype was seen at P2 [*F*(3,100) = 3.810, *p* = 0.012; Figure 5B] with *Peg3KO*^(Mutant litter)^ females calling on average 52 times less than WT^(WT litter)^ females (*p* = 0.008).

These results were independent from body temperature changes before and after testing (Supplementary Table 6), which was included as a covariate in the analyses. In conclusion, loss-of-function of *Peg3* was associated with a deficit in pup communication more apparent in males than females.

### Direct social interaction test

**Figure 6:**
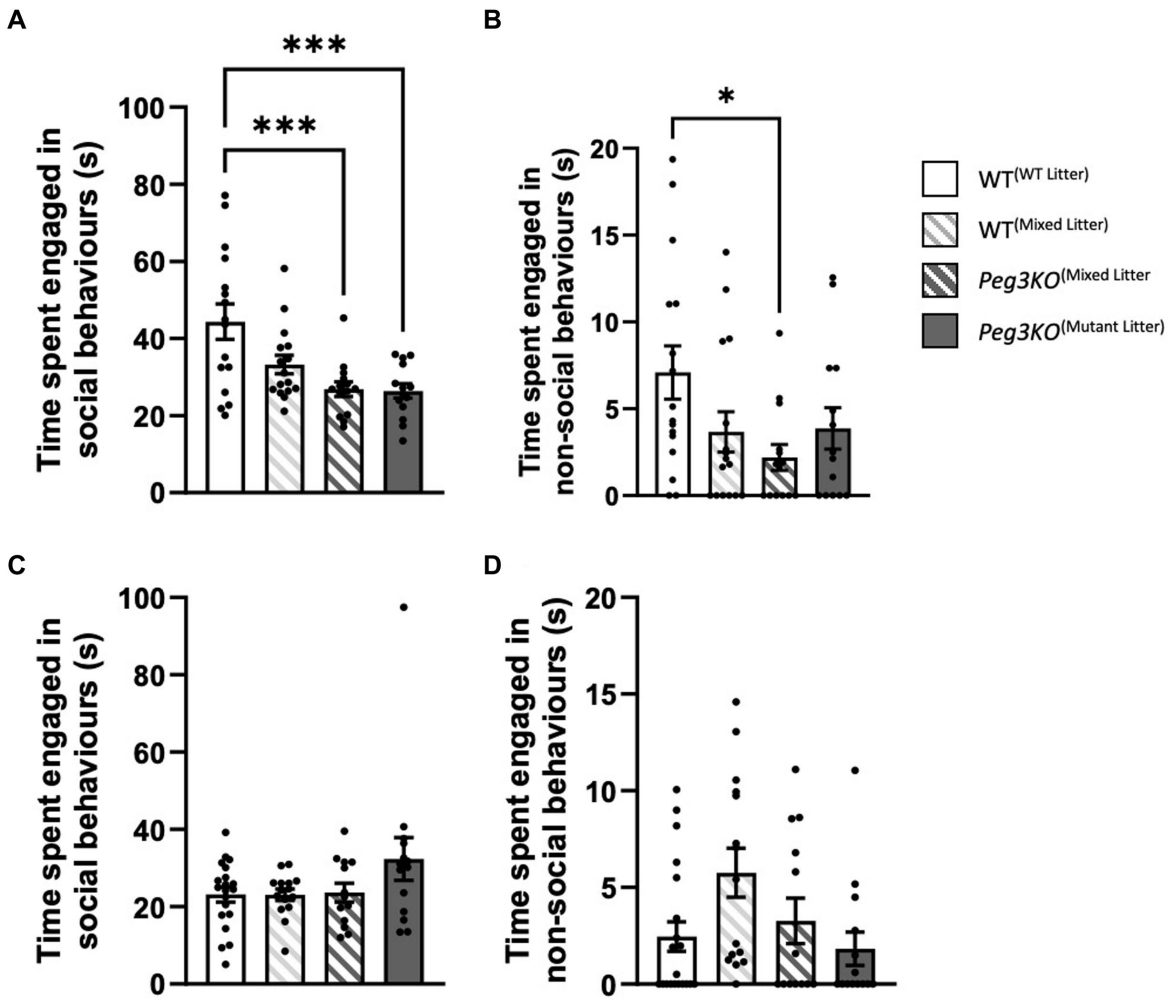
Direct social interaction test. Age = P28. Data is presented from the direct social interaction task for males **(A,B)** and females **(C,D)**. Panels **(A,C)** show the average time spent engaged in social behaviour. A one-way ANOVA showed that mutant males from both single and mixed genotype litters spent significantly less time engaged in social behaviour than WT males from a single genotype litter. **(B,D)** Average time engaged in non-social behaviour. Mutant males from mixed genotype litters spent less time engaged in non-social behaviour than WT males from single genotype litters. No difference were observed between females. Number of offspring: WT^(WT Litter)^ M = 16, F = 20; WT^(Mixed Litter)^ M = 16, F = 15; *Peg3KO*^(Mixed Litter)^ M = 13, F = 13, and *Peg3KO*^(Mutant Litter)^ M = 14, F = 14. Data are mean +SEM. ^*^p < 0.05, ^***^p < 0.001.

A number of tests were used to interrogate post-weaning social behaviours. The direct social interaction test was used to measure the tendency of a mouse to interact with an unfamiliar female mouse of a different strain to the test subject prior to sexual maturity. For males, significant differences were observed in both social and non-social behaviour [Genotype: *F*(3,55) = 1.22, *p* ≤ 0.01] and [*F*(3,55) = 2.83, *p* = 0.05, respectively; Figures 6A,B]. *Peg3KO*^(mutant litter)^ males and *Peg3KO*^(mixed litter)^ males engaged in less social behaviour than WT^(WT litter)^ males (*p* < 0.001). *Peg3KO*^(mixed litter)^ males also engaged in less non-social behaviour than WT^(WT litter)^ males (*p* < 0.04). Females consistently showed no effect of genotype on either social [*F*(3,58) = 2.08, *p* = 0.113] or non-social behaviour [*F*(3,58) = 2.82, *p* = 0.05; Figures 6C,D].

### Social propinquity test

**Figure 7:**
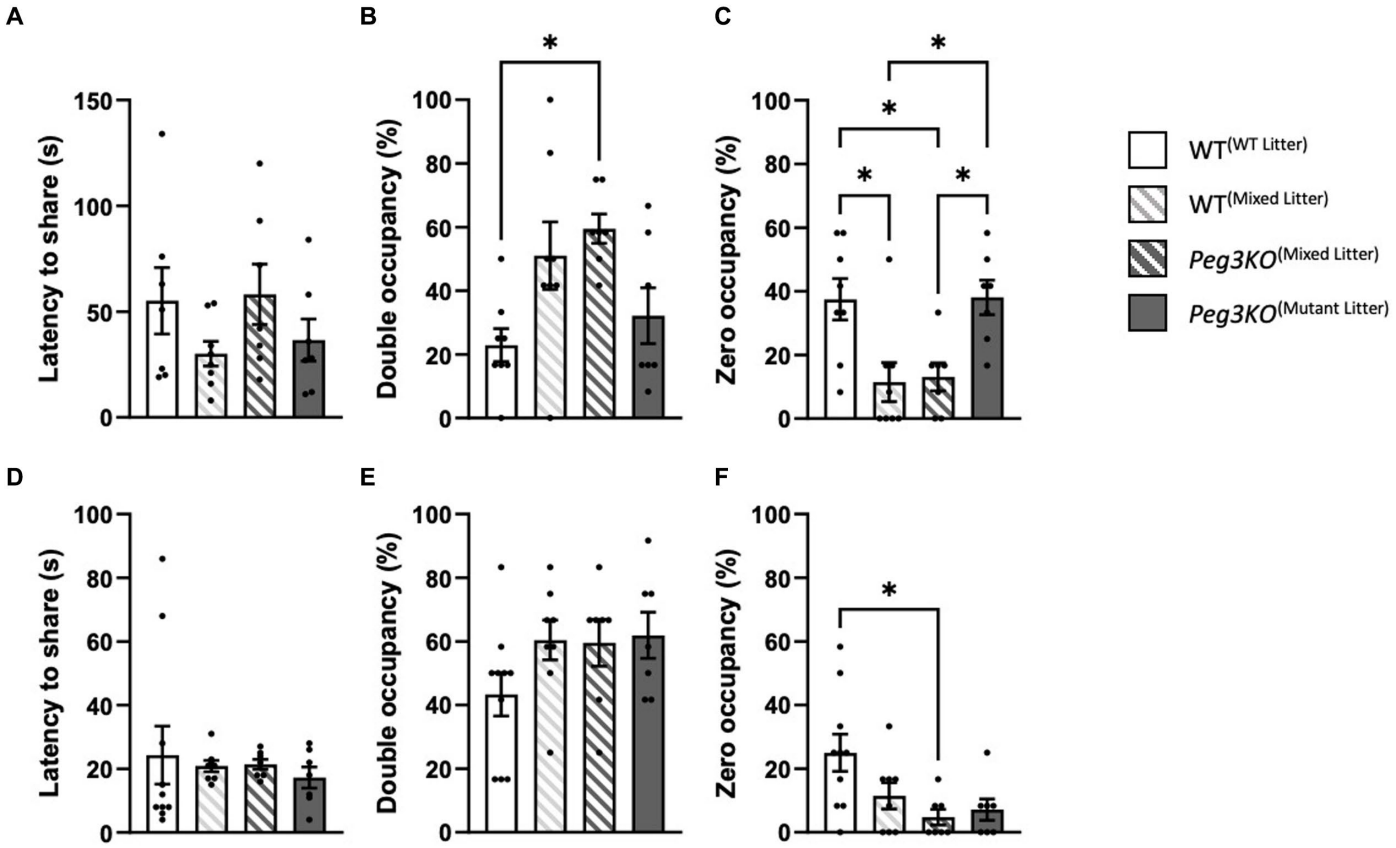
Social propinquity test. Age = P35. Data is presented from the Social Propinquity test for males **(A–C)** and females **(D–F)** analysed using one-way ANOVAS. Panels **(A,D)** show the latency to share the tube. No significant effect of group was detected for males or females. Panels **(B,E)** show the percentage time mice spent sharing the tube. **(B)** *Peg3KO*^(Mixed Litter)^ male mice spent a significantly greater percentage of time sharing the tube than WT^(WT Litter)^. Panels **(C,F)** show percentage of time the tube was vacant. **(C)** Both WT^(Mixed Litter)^ and *Peg3KO*^(Mixed Litter)^ littermate males spent a greater percentage of time in the aversive arena than males from single genotype litters. **(F)** Female *Peg3KO*^(Mixed Litter)^ spent a greater percentage of time in the aversive arena than WT^(WT Litter)^, Number of pairs tested: WT^(WT Litter)^ M = 7, F = 10; WT^(Mixed Litter)^ M = 8, F = 8; *Peg3KO*^(Mixed Litter)^ M = 7, F = 7, and *Peg3KO*^(Mutant Litter)^ M = 7, F = 7. Data are mean ±SEM. ^*^p < 0.05.

The social propinquity test was used to measure anxiety-like and social behaviours by exploring the tendency for mice to maintain close physical proximity to an unfamiliar mouse of the same sex and genotype. In this task, mice choose between sharing a cardboard tube or remaining in the open. For males there were no differences observed in the latency to share the tube [*F*(3,26) = 1.17, *p* = 0.34; Figure 7A]. There was a significant effect of group on percentage time that the tube was shared [*F*(3,26) = 4.62, *p* = 0.01; Figure 7B], with *Peg3KO*^(mixed litter)^ males spending on average 37% more time sharing a tube than WT^(WT litter)^ males (*p* = 0.02). Significant differences were observed in the time the tube was vacant [*F*(3,26) = 6.57, *p* = 0.002; Figure 7C]. The tube was left vacant for less time for both *Peg3KO*^(mixed litter)^ and WT^(mixed litter)^ males compared to both WT^(WT litter)^ and *Peg3KO*^(mutant litter)^. WT^(mixed litter)^ male pairs spent on average 26% (*p* = 0.017) and 27% (*p* = 0.037) less time sharing the tube than WT^(WT litter)^ and *Peg3KO* ^(mutant litter)^ males, respectively. These findings could indicate that mutant male mice from mixed litters were more social than WT males from WT litters, and that males of both genotypes in the mixed litter group were more anxious than males from single genotype litters.

For females, there were no differences in latency to share the tube [*F*(3,28) = 0.32, *p* = 0.87; Figure 7D] or tube sharing [*F*(3,28) = 1.79, *p* = 0.17; Figure 7E] but there was a difference in the time the tube was vacant across the groups [*F*(3,28) = 4.26, *p* = 0.01] with a significant reduction for *Peg3KO*^(mixed litter)^ compared to WT^(WT litter)^ females (*p* = 0.02; Figure 7F).

### Three-chamber test

**Figure 8:**
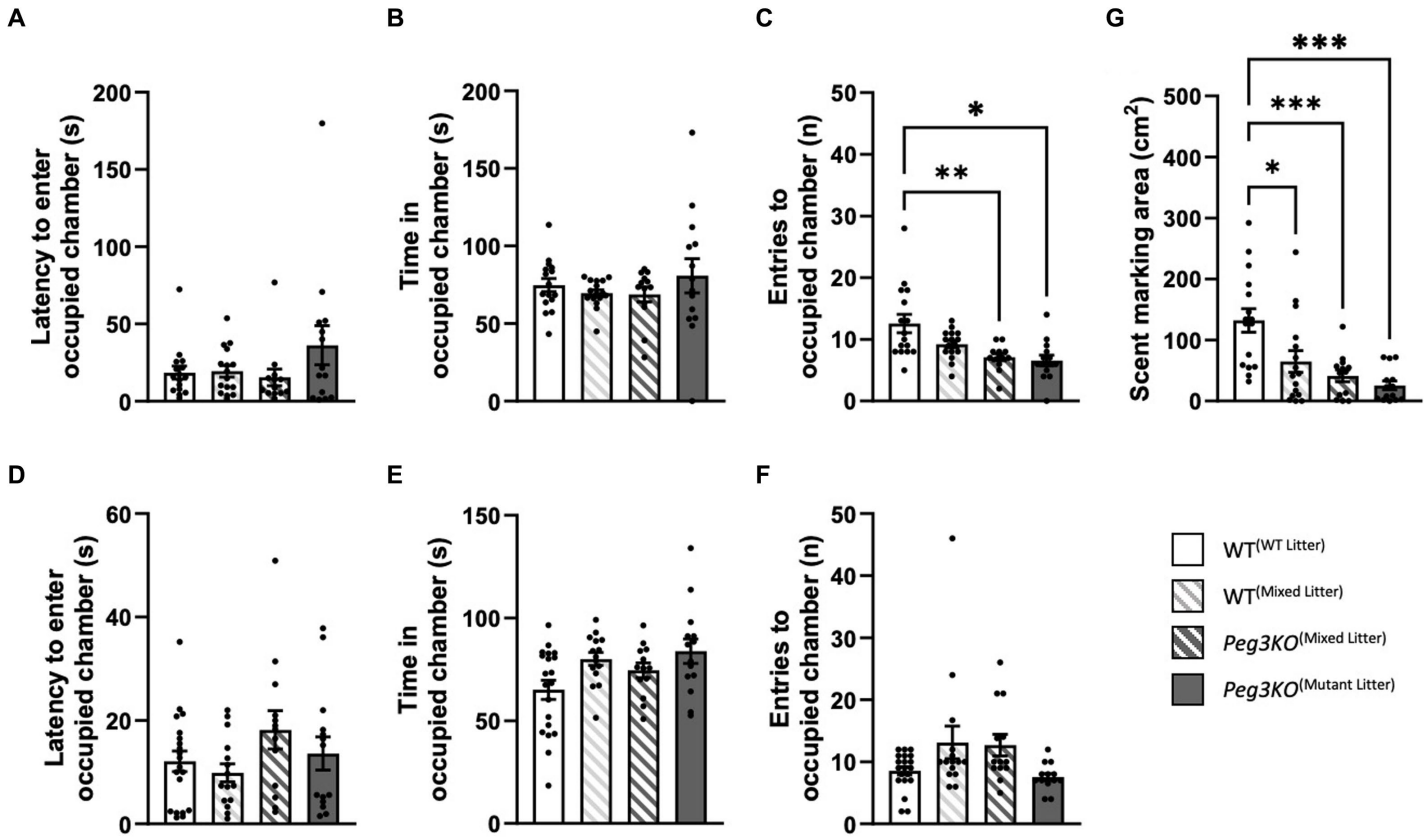
Three-chamber test and scent marking test. Age = P63. Data is presented for males **(A–C,G)** and females **(D–F)**. All data were analysed using one-way ANOVAs. Panels **(A,D)** show the latency to enter the occupied chamber. Panels **(B,E)** show the total duration spent in the occupied chamber. Panels **(C,F)** show the number of entries made into the occupied chamber. WT^(mixed litter)^ males made a greater number of crosses into the occupied chamber than both *Peg3KO*^(Mixed Litter)^ (*p* = 0.006) and *Peg3KO*^(Mutant Litter)^ (*p* = 0.03) litter. Panel **(G)** shows the total number of 1 cm² squares containing scent markings made during the three-chamber trial (males only). All experimental groups scent marked less that WT^(WT Litter)^ males. Number of offspring: WT^(WT Litter)^ M = 16, F = 20; WT^(Mixed Litter)^ M = 16, F = 15; *Peg3KO*^(Mixed Litter)^ M = 13, F = 13, and *Peg3KO*^(Mutant Litter)^ M = 14, F = 14. Data are mean ±SEM. ^*^p < 0.05, ^**^p < 0.01, ^***^p < 0.001.

A three-chamber test was used to assess general sociability and interest in social novelty by providing an unfamiliar mouse of the opposite sex and a different strain. No effect of genotype was observed on the latency to enter the occupied chamber for males [*F*(3,55) = 2.55, *p* = 0.65; Figure 8A]. Whilst there was a significant effect of genotype in males on time spent in the occupied chamber [*F*(3,55) = 3.63, *p* = 0.02; Figure 8B], *post-hoc* results did not survive Bonferroni correction with no group differences achieving statistical significance. There was difference in the number of entries into the chamber containing the unfamiliar host [*F*(3,58) = 3.57, *p* = 0.02; Figure 8C], with *Peg3KO*^(mixed litter)^ and *Peg3KO*^(mutant litter)^ males both crossing on average 5 fewer times than WT^(WT litter)^ males (*p* = 0.006 and *p* = 0.03, respectively). For females, there were no significant findings latency [*F*(3,58) *p* = 0.20; Figure 8D], time [*F*(3,58) = 0.08, *p* = 0.97; Figure 8E] or entries [*F*(3,55) = 0.77, *p* = 0.52; Figure 8F].

### Scent marking

Scent marking was assessed in males during the three-chamber test and a significant effect of genotype was apparent [*F*(3,55) = 9.63, *p* < 0.001; Figure 8G]. Compared to WT^(WT litter)^, WT^(mixed litter)^ males covered on average 67 cm^2^ less (*p = 0*.01), *Peg3KO*^(mixed litter)^ (*p* < 0.001) 91 cm^2^ less and *Peg3KO*^(mutant litter)^ (*p* < 0.001) covered 107 cm^2^ less area in urine suggestive of decreased territorial behaviour or social dominance.

### Courtship USVs

**Figure 9:**
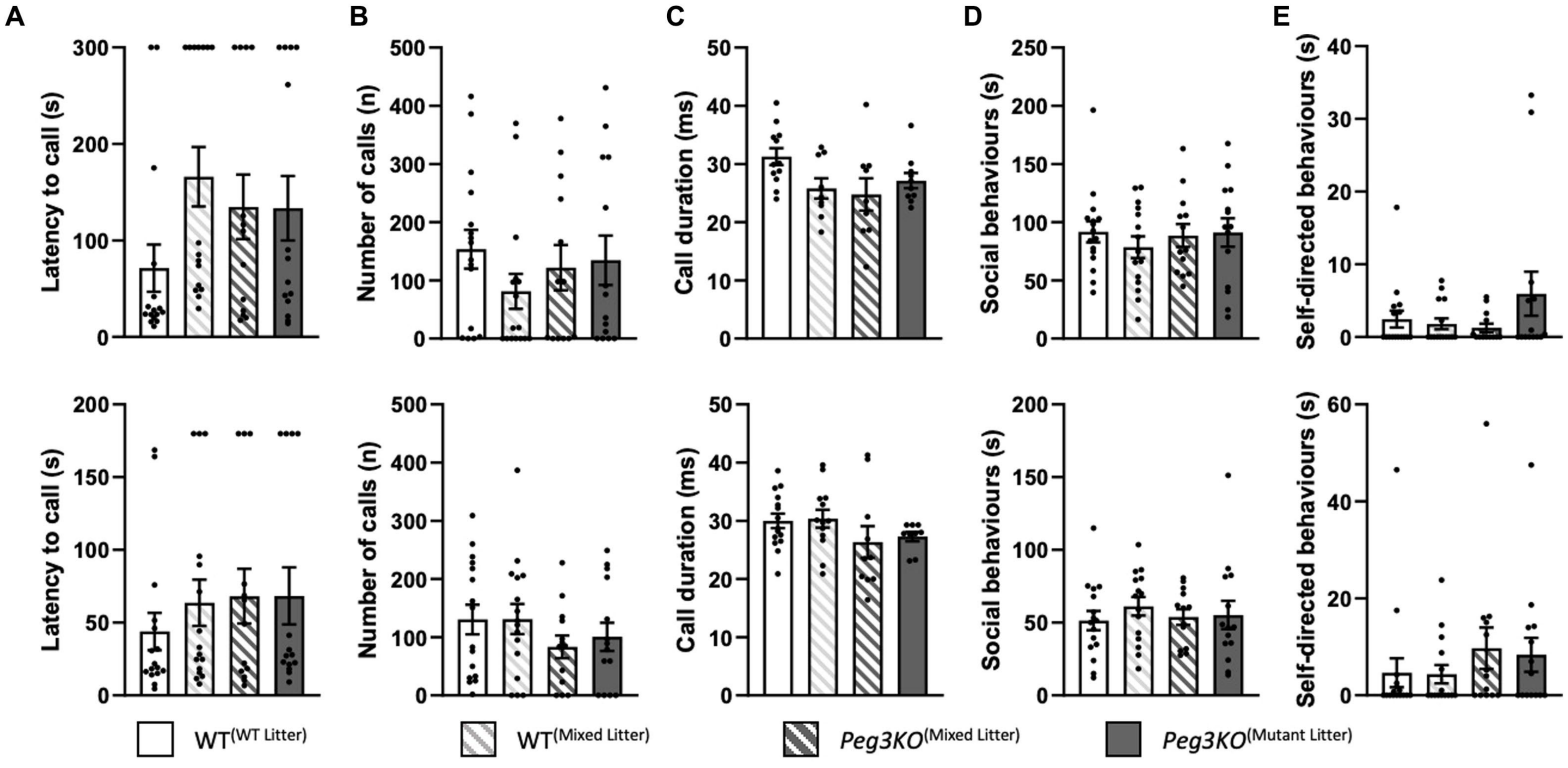
Courtship USVs and behaviours. Age = P70. Male mice during interaction with a female WT mouse in oestrous—initial phase data (above) and reunion phase data (below). **(A)** Latency to call. **(B)** Total number of calls. **(C)** Mean duration of call. **(D)** Time spent engaged in social behaviour. **(E)** Time spent engaged in self-directed behaviour. No effect of genotype was detected for any of the USV parameters in either phase. Number of offspring: WT^(WT Litter)^ M = 16; WT^(Mixed Litter)^ M = 16; *Peg3KO*^(Mixed Litter)^ M = 13, and *Peg3KO*^(Mutant Litter)^ M = 14. Data are mean ±SEM.

Courtship USVs by males were tested in two phases. There was no significant effect of genotype on latency to call, number of calls or mean duration of call in the initial phase [*F*(3,55) = 1.87, *p* = 0.15 and *F*(3,55) = 0.71, *p* = 0.55 and *F*(3,55) = 1.24, *p* = 0.300, respectively; Figures 9A–C, upper panels] or the reunion phase [*F*(3,55) = 0.50 and *p* = 0.68; *F*(3,55) = 0.79, *p* = 0.50 and *F*(3,55) = 2.00, *p* = 0.23, respectively; Figures 9A–C, lower panels]. There was no significant differences in time spent engaged in social behaviour or non-social behaviour in the initial phase [*F*(3,55) = 0.74 *p* = 0.53 and *F*(3,55) = 1.60 *p* = 0.20, respectively; Figures 9D,E, upper panels] or the reunion phase [*F*(3,55) = 0.12, *p* = 0.95 and *F*(3,55) = 0.71, *p* = 0.55, respectively; Figures 9D,E, lower panels].

## Discussion

In this study, we reproduced our original finding that WT dams carrying and caring for *Peg3*-deficient offspring were slower to retrieve their mutant pups and exhibited increased anxiety-like behaviour compared to WT dams with WT pups (McNamara et al., 2018) demonstrating the robustness of this finding. We further identified a reduction in pup-directed behaviour by WT mothers towards their mutant pups. USV analysis of individual pups uncovered a male-biased communication deficit in mutant offspring 4 to 6 days after birth. As young adults, *Peg3* mutant males demonstrated abnormal behaviours in a number of sociability tests, whereas female mutants generally did not. These sexually dimorphic behavioural outcomes support previous findings that imprinted genes function both in a parent-of-origin manner and in a manner dependent on the sex of the individual.

Previous research reports that loss of *Peg3* expression is associated with *in utero* growth restriction and lighter placenta (Li et al., 1999; Curley et al., 2004; Kim et al., 2013; Denizot et al., 2016). We reported a severe placental endocrine lineage defect more apparent in mutant male placenta compared to female (Tunster et al., 2018). In this present study both male and female *Peg3KO* mutant offspring were significantly lighter than WT controls when first weighed 2 days after birth irrespective of their shared environment. Low pup weight very early in life could be caused by placental insufficiency or intrinsic growth restriction. Previous studies have reported sustained growth restriction into adulthood when comparing mutant animals with WT littermates (Kim et al., 2013; Denizot et al., 2016; Tanaka et al., 2022). We similarly observed that male and female mutant *Peg3KO*^(mixed litter)^ pups sharing their environment with WT siblings were growth restricted through lactation and after the mice were weaned. Again, sustained growth restriction could be interpreted to reflect either the reduced ability of mutants to effectively compete for resources and/or intrinsic growth restriction. However, in our study the genetically identical *Peg3KO*^(mutant litter)^ mice in 100% mutant litters exhibited catch up growth achieving a comparable weight to the fully WT males and females as early as P4 and P6, respectively. Our results show that growth restriction only continues when there is competition with WT littermates – both during lactation and postweaning—supporting extrinsic growth restriction due to competition. These patterns of catch-up vs. sustained growth restriction early in life may contribute to different outcomes later in life for *Peg3KO* mutant mice.

Previously we reported that dams carrying and caring for litters consisting entirely of mutant pups displayed anxiety-like behaviour using just the EZM test. Here we applied the EZM at P4 and the LDB test at P7 to validate and extend our original findings with the inclusion of litters where only half the pups were mutant. Whilst there were no significant differences in the Dam^(mutant litter)^ behaviour in three traditional assessments of anxiety-like behaviour used in the EZM test (time spent in the open section, latency to enter the open section and the frequency of crosses), significant differences were observed in the frequency of stretch-attend postures indicating an anxiety-related avoidance in passive exploratory-anxiety conflict (Rey et al., 2012). In the LDB test Dam^(mutant litter)^ demonstrated an increased latency to enter the light section of the arena and a greater number of stretch-attend postures than Dam^(WT litter)^. Furthermore, Dam^(mixed litter)^ spent less time in the light section than Dam^(WT litter)^. Both the EZM and LDB measure anxiety-like behaviours (Ramos et al., 1997). However, the psychological aspects of the different tests (e.g., aversion to light vs. aversion to open spaces/falling) are not identical (Ramos, 2008), and the LDB test presents a more subtle anxiogenic environment compared to EZM. The findings across both tests provides a more robust assessment of anxiety further evidenced using a unified maternal anxiety score generated by combining the scores from all anxiety-related measures within the EZM and LDB (Supplementary Table 3). Using the unified score, Dam^(mutant litter)^ displayed a higher maternal anxiety score than Dam^(WT litter)^, consistent with our previous study (McNamara et al., 2018). These data show a significant increase in anxiety-like behaviours in our dams that carried litters composed entirely of *Peg3KO* offspring, compared to those with all WT. Dams of mixed genotype litters spent less time in the light in the LDB and generally scored in between Dam^(WT litter)^ and Dam^(mutant litter)^ in most other measures suggesting that the presence of WT pups was not sufficient to fully counteract the impact of the mutant pups. Given that all dams were genetically WT, these results further demonstrate that maternal anxiety traits are caused by the reduced dosage of *Peg3* in the offspring.

In the current study we assessed pup retrieval on P3 so as not to conflict with the recording of individual pup USVs made on P2. We analysed the average time to retrieve each pup by sex and genotype to ensure that both sexes and all genotypes were represented when analysing maternal responsiveness. This revealed a delay in the retrieval of *Peg3*-deficient pups in all-mutant litters relative to all-WT litters, essentially consistent with our previous study at P2 (McNamara et al., 2018), and an intermediate retrieval time for pups in mixed litters of both genotypes. We were not able to detect a significant discrimination between male and female pups of the same genotype nor were dams able to discriminate between mutant and WT males in our study. However, due to considerable variation in this measure we cannot exclude a more subtle phenotype. As in previous studies (Li et al., 1999), maternal behaviour was assessed during the pup retrieval task. During pup retrieval there is a conflict between the time spent retrieving each pup, the time spent nurturing the retrieved pups and self-directed behaviour. Dams^(mutant litter)^ were found to spend significantly less time engaged in pup directed behaviour than Dams^(WT litter)^ and Dams^(mixed litter)^ supporting the conclusion that reduction in offspring *Peg3* expression disrupts the provision of postnatal maternal care. We did not detect significant changes with respect to pup retrieval or pup-directed behaviour in dams of mixed litters compared to those with fully WT litters although the intermediate phenotype is consistent with a dosage-effect. It was not possible to discern whether there was preferential allocation of maternal care towards a particular genotype or sex due to pups being hidden in the nest. By age P9, differences in maternal care were attenuated, likely because maternal behaviour becomes less intense as rodent offspring age (Champagne et al., 2007).

Pup retrieval and care is not a passive process for neonatal pups. Pup USVs are emitted from birth to attract the attention of their mothers, and encourage their retrieval when displaced from the nest (Champagne et al., 2007). USV emission typically follows a ‘U-shaped’ developmental trajectory, peaking around day 6–8 before reducing in number. Previously we observed that a group of four *Peg3KO*^(mutant litter)^ pups emitted significantly fewer USVs than WT^(WT litter)^ immediately prior to the pup retrieval task (McNamara et al., 2018). In the current study we did not detect this significant difference which may be due methodological differences. Notably, in the current study, rather than being tested in the home cage as previously (McNamara et al., 2018), USVs were recorded within an isolation chamber to reduce background noise. However, when tested as individuals using the latter method, we observed that *Peg3KO*^(mutant litter)^ males called significantly less than WT^(WT litter)^ males at both P4 and P6. Furthermore, whilst we observe the expected call number over time in the WTs with both males and female increasing in number over time, USV emissions in the male *Peg3KOs*^(mutant litter)^ followed a more flattened trajectory, increasing only negligibly before dropping off at P10. In contrast, *Peg3KO*^(mutant litter)^ females called significantly less than WT^(WT litter)^ females only at P2. This deficit was not attributed to a drop in body temperature during testing, a factor which has been shown to effect USV emission (Ehret, 2005). Previous studies on both non-imprinted genes such as *Foxp1* (Frohlich et al., 2017) and imprinted genes such as *Magel2* (Bosque Ortiz et al., 2022) report pup USV deficits but not with a more noticeable impact on males. In general, wildtype male pups vocalise significantly more than wildtype female pups (Frohlich et al., 2017) with similar findings in our study (Supplementary Figure 6). Whilst we were not able to detect a difference in maternal behaviour relative to either the sex or the genotype of the pup, male pups may generally be more demanding of maternal attention than female pups, with loss of *Peg3* function attenuating male demands.

Whilst there is some debate as to the function of USVs by pups over time, in adult mice and between males and females (Portfors, 2007), vocalisation is a key element of social behaviour. Therefore, our post-weaning behavioural analysis focused primarily on assessing sociability. In each of these tests the experimental animals were challenged with unfamiliar animals to tease out different aspects of behaviour. In the direct social interaction test, where the test mouse was observed interacting with an unfamiliar female mouse of a different strain, significant differences between groups were observed for males. Specifically, *Peg3* mutant males spent less time engaging in social behaviour irrespective of whether they were from mixed or 100% mutant litters. *Peg3KO*^(mixed litter)^ males also spent less time engaged in non-social behaviours. There were no significant difference between female groups. Overall, females spent more time exploring the arenas than males with a reduced percentage time engaged in social/self behaviours consistent with previous studies reporting that male mice investigate conspecific strangers more than females (Karlsson et al., 2015) and females explore novel arenas and objects more than males (Bettis and Jacobs, 2009) potentially influencing the findings in females. However, there was also a male-bias in findings in the social propinquity test, where the test mouse was observed interacting with an unfamiliar mouse of the same sex and genotype. Both WT^(mixed litter)^ and *Peg3KO*^(mixed litter)^ males spent more time sharing the tube with unfamiliar males of the same genotype compared to WT^(WT litter)^ males. Essentially, these mice are choosing the share the shelter over being out in the open space or alone in the tube suggestive of increased sociability. *Peg3* mutant males raised in all mutant litters essentially behaved like WT controls suggesting growing up in a mixed litter contributed to this phenotype. The only significant difference observed in females was that the tube was empty for less time by *Peg3KO*^(mixed litter)^ females. In the three-chambers test, where test mice are faced with an unfamiliar mouse of a different strain and opposite sex, there were no differences in latency to enter the occupied chamber or the time spent in the occupied chamber but both *Peg3KO*^(mixed litter)^ and *Peg3KO*^(mutant litter)^ males entered the chamber fewer times than WT^(WT litter)^ males. No differences were detected for females. Compared to the control WT^(WT litter)^ males, all three experimental groups of males engaged in less scent marking behaviour suggestive of reduced social dominance as previously reported using alternative tests (Tanaka et al., 2022). During the courtship test, no significant differences were detected. However, USV differences may have been masked by the substantial variation between individual animals both in latency to call and the number of calls. In summary, *Peg*3 mutant males demonstrated early life deficits in communication and later life deficits in social behaviours across a number of tests whereas female mutants were less affected.

In some tests there were differences in the findings for mutant males depending on whether they were raised in mixed or single genotype litters. For example, in the social propinquity test social deficits were observed in *Peg3KO*^(mixed litter)^ males but not *Peg3KO*^(mutant litter)^ males. In this test WT^(mixed litter)^ males who shared the pre-and postnatal environment with mutant males also showed deficits. Similarly, WT^(mixed litter)^ males scent marked less than controls. This reveals that behavioural changes in males were not solely driven by intrinsic loss of *Peg3* function. WT^(mixed litter)^ males may behave differently as a result of being raised by a dam with behavioural deficits (anxiety, pup care) or due to their interaction during development with littermates who have social deficits or due to different postnatal growth dynamics. Whatever the reason for these differences, this work provides another example where WT mice sharing their early life environment with genetically altered mice behave in an abnormal manner when compared to a fully controlled scenario (Sledziowska et al., 2020; Harrison et al., 2021) and highlights an often overlooked component in experimental design using genetic models.

Pup USVs cannot be directly compared to human communication (Portfors and Perkel, 2014) but they have been used to assess developmental delays and communication deficits in rodent models of disorders such as autistic spectrum disorder (Gal et al., 2023). Similarly, the altered social behaviours we have observed in mice are not directly comparable to behavioural disorders observed in human children. Nonetheless, our findings may shed mechanistic insight on the reported association of maternal mood symptoms with delays in language development and behavioural disorders most predominantly observed for boys (Van den Bergh et al., 2005; Talge et al., 2007; Glover and Hill, 2012; Raikkonen et al., 2015; Lahti et al., 2017; Savory et al., 2020; Zhang et al., 2023). We previously reported lower *PEG3* expression in the male term placenta of women suffering depression in pregnancy—both those clinically diagnosed and those with questionnaire-identified depressive symptoms (Janssen et al., 2016a) which could be interpreted to mean that mood symptoms cause lower *PEG3* expression in offspring. However, in our ‘preclinical’ model of reduced expression of *PEG3* confined to the offspring we observe anxiety in WT mothers, and communication and social deficits in male offspring proving that these seemingly independent outcomes for mothers and their sons can be caused from the same underlying molecular defect, at least in mice.

In summary, we report male-biased pup USV deficits and atypical social behaviour in *Peg3KO* mice providing further evidence that imprinted genes can have a sexually-dimorphic impact on phenotype. Given the finding that the dosage of *Peg3* in the offspring also influences maternal anxiety-like behaviour, it is possible that reduced expression of *PEG3* in human offspring underlies the co-occurrence of maternal mood disorders and atypical male-specific behaviours observed in human children.

## Data availability statement

The raw data supporting the conclusions of this article will be made available by the authors, without undue reservation.

## Ethics statement

The animal study was approved by University of Cardiff Ethical Committee and performed under a UK Home Office project license. The study was conducted in accordance with the local legislation and institutional requirements.

## Author contributions

HT: Conceptualization, Formal analysis, Investigation, Methodology, Writing – review & editing. DH: Methodology, Writing – review & editing, Investigation. MH: Investigation, Methodology, Writing – review & editing. AI: Conceptualization, Methodology, Supervision, Writing – review & editing. RJ: Conceptualization, Funding acquisition, Project administration, Supervision, Writing – original draft, Writing – review & editing.
