# Supplementary material for: Deficiency of the paternally-expressed imprinted Peg3 gene in mice has sexually dimorphic consequences for offspring communication and social behaviour
Source: Front Neurosci. 2024 Mar 26;18:1374781. doi: 10.3389/fnins.2024.1374781 (PMC11002209; doi:10.3389/fnins.2024.1374781)
Supplement: Supplementary file 1 [file Data_Sheet_1.docx]

**Supplementary Tables**

**Supplementary Table 1. Dam behaviours observed during the pup retrieval task that were used to calculate the time spent engaged in ‘Pup-directed’ and ‘Non-pup directed’ behaviour.**

| **Behaviours** | **Definition** | **Composite variable category** |
| --- | --- | --- |
| Exploring | Exploring the home cage, not engaging with pups or the nest, e.g., digging, running around | Non-pup directed |
| In nest | In the nest but without pups, or can be seen not to be engaged in pup-directed behaviour whilst in the nest | Non-pup directed |
| Sniff pup | Sniffing, or nose contact with the pup | Pup directed |
| Retrieval attempt | The dam attempts to move the pup toward the nest, actively picking up the pup in her mouth, or handling the pup with her forepaws whilst moving toward the nest | Pup directed |
| Groom pup | Dam touches the pup’s body with her tongue, or the dam handles the pup’s body with her forepaws or nose | Pup directed |
| Crouching | Dam is in the nest and crouched over pups with little to no movement. If partially obscured by the nest, dam is only assumed to be crouching if pups are present in the nest and there is little movement | Pup directed |
| Nest build | Collecting and / or handling nesting material with mouth or forepaws | Pup directed |
| Self-groom | Self-grooming whiskers or body | Non-pup directed |
| Freeze | Not moving, stationary, whilst not engaging with pups or nest | Non-pup directed |

**Supplementary Table 2. Variables used to calculate the time spent engaged in ‘Social’ and ‘Non-social’ behaviours during the Direct Social Interaction and Courtship behaviour tests*.**

| **Social Interaction** | **Non-social behaviour** |
| --- | --- |
| Nose-to-nose sniffing | Grooming self |
| Ano-genital sniffing | Immobile |
| Body sniffing |  |
| Following |  |
| Being followed |  |
| Being attacked |  |
| Mounting |  |

*As in Harrison *et al*. ^44^ exploring was not included in either social or non-social behaviour

**Supplementary Table 3. Anxiety score:** **Measures of anxiety used to calculate the unified maternal anxiety score and direction of influence.**

| **Test** | **Measure** | **Influence** | **Reference** |
| --- | --- | --- | --- |
| Elevated Zero Maze | Number of crosses | -ve | ^51,52^ |
|  | Time in open | -ve | ^51-54^ |
|  | Latency to enter open | +ve | ^51,52^ |
|  | Frequency of stretch-attend posture | +ve | ^55-57^ |
| Light Dark Box | Number of crosses | -ve | ^58,59^ |
|  | Time in light | -ve | ^54,58,59^ |
|  | Latency to enter light | +ve | ^58,59^ |
|  | Frequency of stretch-attend posture | +ve | ^55-57^ |

**Supplementary Table 4.** Mean percentage of time that dams were engaged in observed behaviours during pup retrieval task at P3. Data are Mean ±SEM.

|  | **Exploring** | **In Nest** | **Sniff pup** | **Retrieval Attempt** | **Groom Pup** | **Crouching** | **Nest Build** | **Self Groom** | **Freeze** |
| --- | --- | --- | --- | --- | --- | --- | --- | --- | --- |
| **Dam^(WT Litter)^** | 46.66  (4.33) | 15.37  (2.63) | 9.49  (1.57) | 11.48  (2.08) | 1.88  (0.76) | 9.51  (2.86) | 4.78  (2.04) | 0.00  (0.00) | 0.83  (0.83) |
| **Dam^(Mixed litter)^** | 55.36  (5.34) | 11.08  (1.63) | 8.34  (1.35) | 10.30  (1.79) | 1.20  (0.64) | 9.67  (4.17) | 2.60  (1.04) | 1.23  (0.72) | 0.22  (0.16) |
| **Dam^(Mutant litter)^** | 56.22  (4.38) | 20.97  (3.85) | 5.62  (0.57) | 10.16  (0.77) | 0.00  (0.00) | 4.48  (2.19) | 1.30  (0.33) | 1.14  (1.14) | 0.11  (0.07) |

**Supplementary Table 5.** Mean percentage of time that dams were engaged in observed behaviours during pup retrieval task at P9. Data are Mean ±SEM.

|  | **Exploring** | **In Nest** | **Sniff pup** | **Retrieval Attempt** | **Groom Pup** | **Crouching** | **Nest Build** | **Self Groom** | **Freeze** |
| --- | --- | --- | --- | --- | --- | --- | --- | --- | --- |
| **Dam^(WT Litter)^** | 65.20  (4.67) | 6.34  (2.05) | 12.26  (5.61) | 13.26  (1.79) | 0.00  (0.00) | 0.14  (0.08) | 2.07  (0.97) | 0.73  (0.73) | 0.00  (0.00) |
| **Dam^(Mixed litter)^** | 60.81  (4.07) | 16.80  (2.37) | 4.46  (0.86) | 10.41  (1.1.8) | 0.02  (0.02) | 1.35  (0.81) | 2.96  (1.25) | 3.19  (1.95) | 0.00  (0.00) |
| **Dam^(Mutant litter)^** | 61.74  (4.68) | 17.94  (3.65) | 6.33  (0.88) | 11.48  (1.44) | 0.00  (0.00) | 0.29  (0.17) | 0.50  (0.49) | 0.36  (0.17) | 0.00  (0.00) |

**Supplementary Table 6.** Average change in body temperature from pre- to post testing during isolation induced USVs data are mean + SEM

|  |  | **P2** | **P4** | **P6** | **P8** | **P10** |
| --- | --- | --- | --- | --- | --- | --- |
| **Male** | WT | 2.34 (0.17) | 1.87 (0.16) | 1.43 (0.14) | 1.12 (0.13) | 1.25 (0.15) |
|  | WT (Mixed litter) | 2.02 (0.26) | 2.17 (0.27) | 1.42 (0.16) | 1.07 (0.17) | 2.07 (0.58) |
|  | *Peg3KO* (Mixed litter) | 2.46 (0.20) | 1.92 (0.22) | 1.74 (0.19) | 1.38 (0.20) | 0.84 (0.16) |
|  | *Peg3KO* (Mutant litter) | 2.57 (0.23) | 1.92 (0.25) | 1.35 (0.15) | 1.06 (0.12) | 1.19 (0.17) |
| **Female** | WT | 2.29 (0.18) | 1.84 (0.15) | 1.40 (0.17) | 1.27 (0.12) | 0.9 (0.14) |
|  | WT (Mixed litter) | 2.69 (0.31) | 3.94 (1.83) | 1.9 (0.25) | 0.94 (0.27) | 0.96 (0.16) |
|  | *Peg3KO* (Mixed litter) | 2.45 (0.23) | 1.91 (0.18) | 1.79 (0.17) | 1.6 (0.21) | 1.00 (0.11) |
|  | *Peg3KO* (Mutant litter) | 3.18 (0.23) | 2.07 (0.24) | 1.59 (0.20) | 1.08 (0.18) | 1.09 (0.15) |
