# Supplementary figures and images for: Deficiency of the paternally-expressed imprinted Peg3 gene in mice has sexually dimorphic consequences for offspring communication and social behaviour
Source: Front Neurosci. 2024 Mar 26;18:1374781. doi: 10.3389/fnins.2024.1374781 (PMC11002209; doi:10.3389/fnins.2024.1374781)

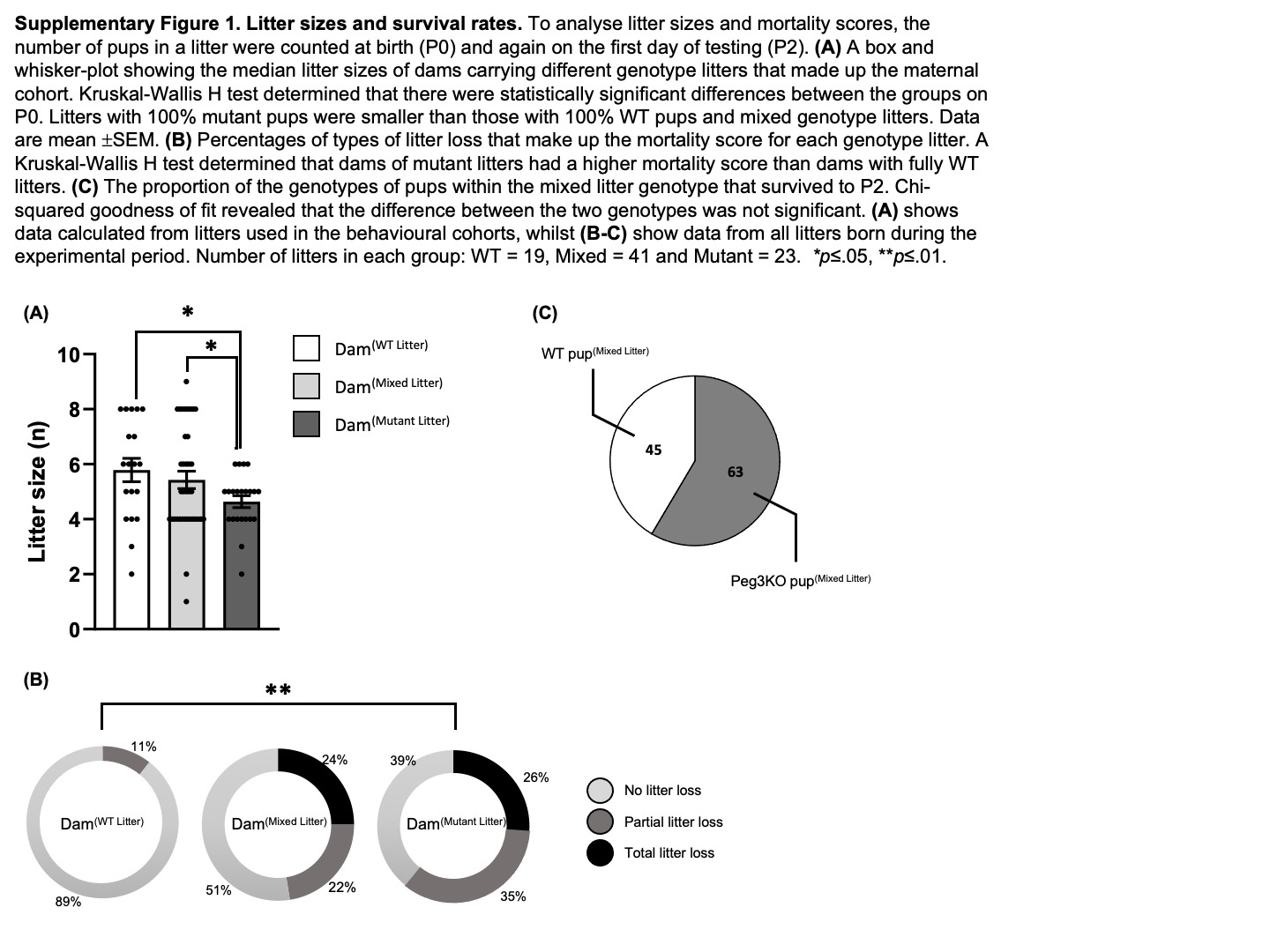

Supplement: Supplementary file 2 [file Image_1.JPEG]

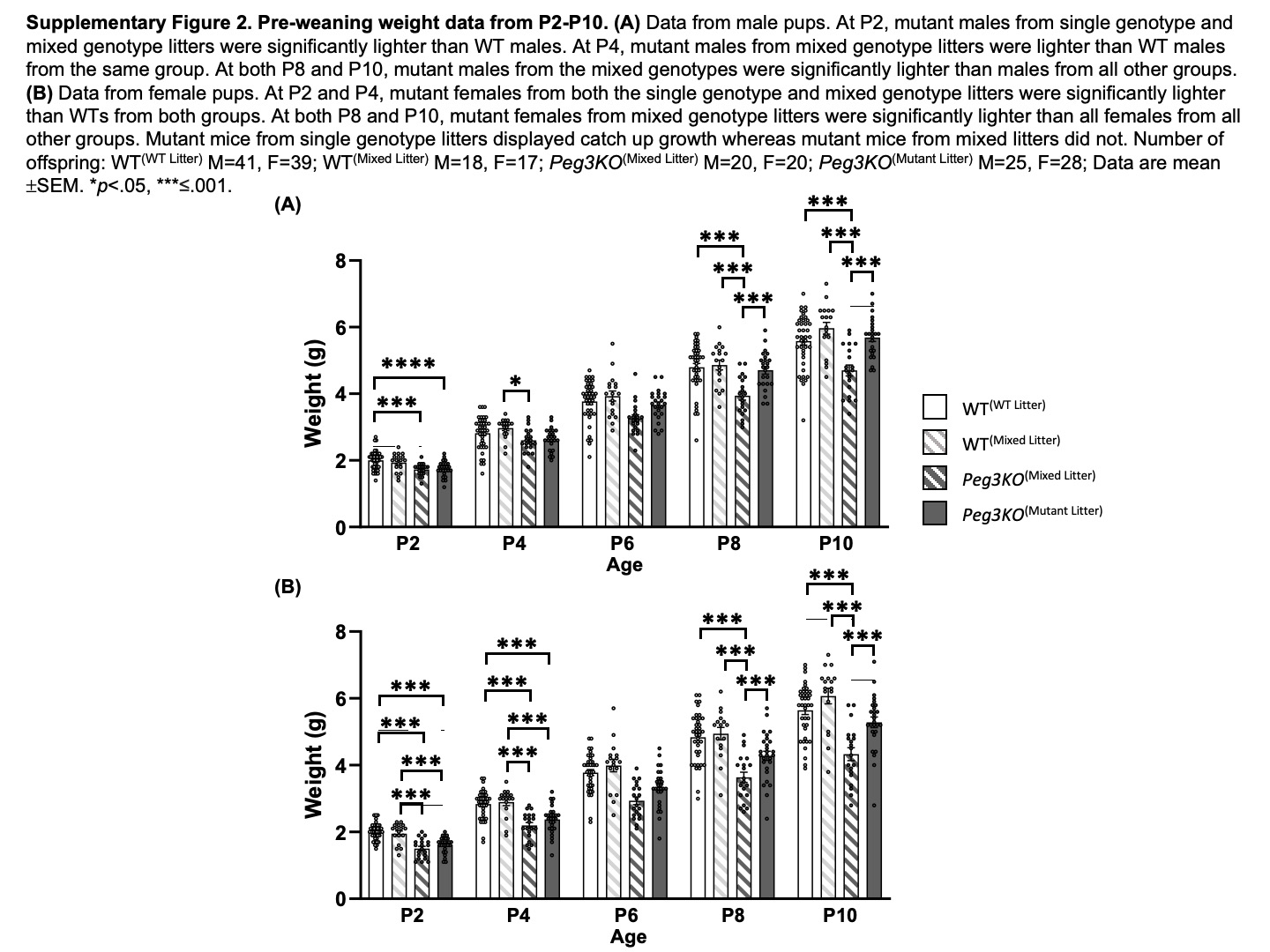

Supplement: Supplementary file 3 [file Image_2.JPEG]

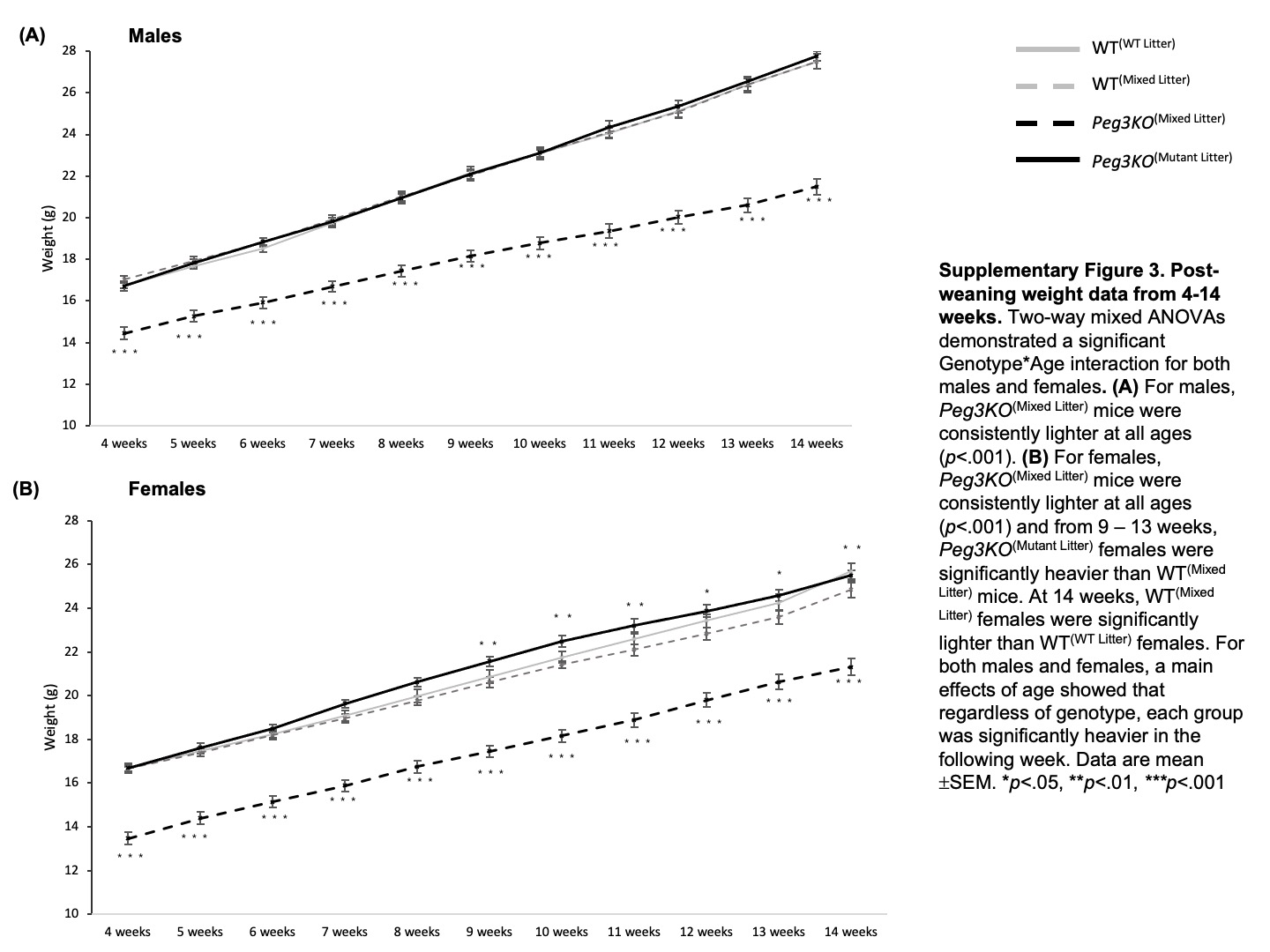

Supplement: Supplementary file 4 [file Image_3.JPEG]

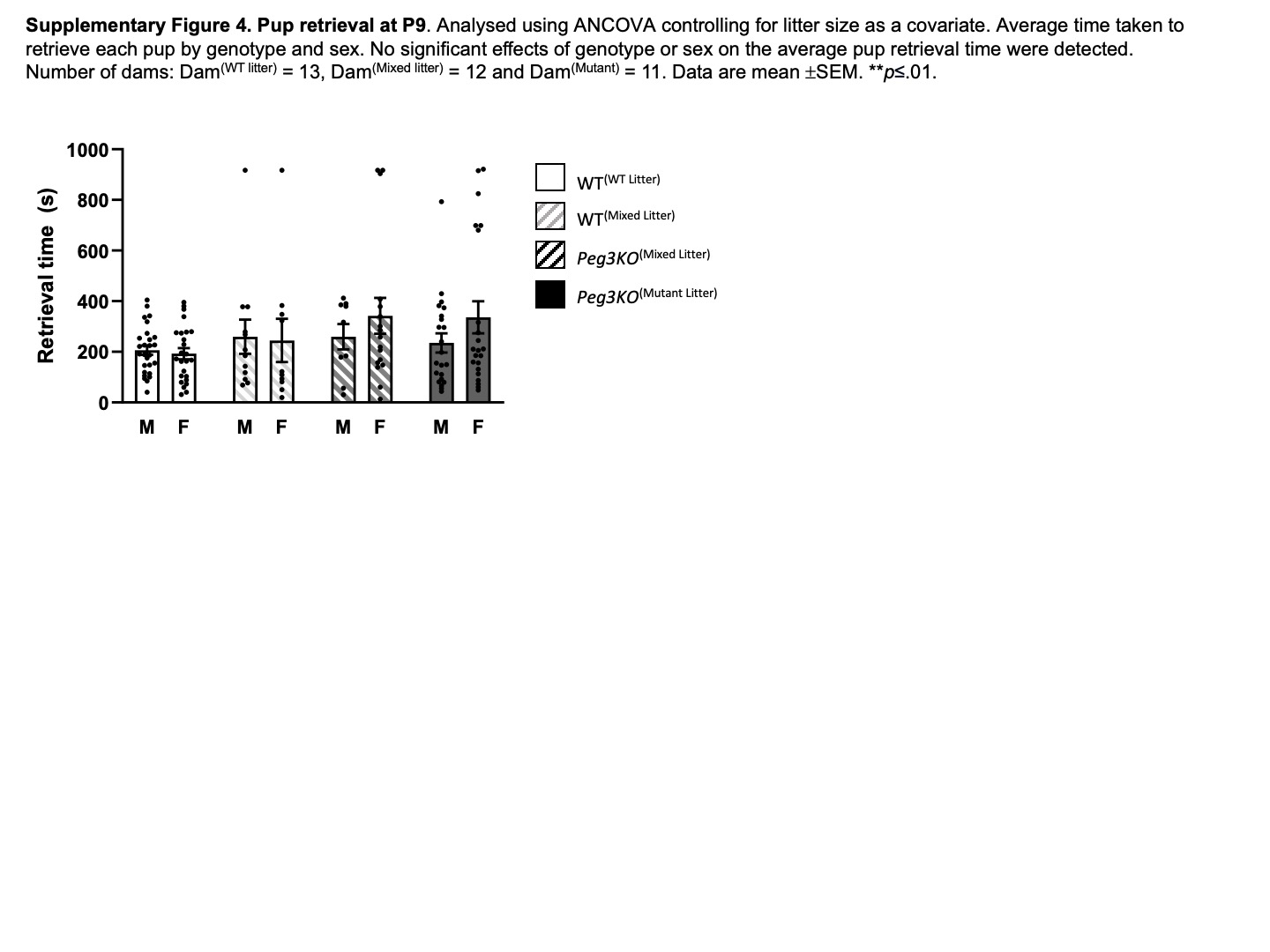

Supplement: Supplementary file 5 [file Image_4.JPEG]

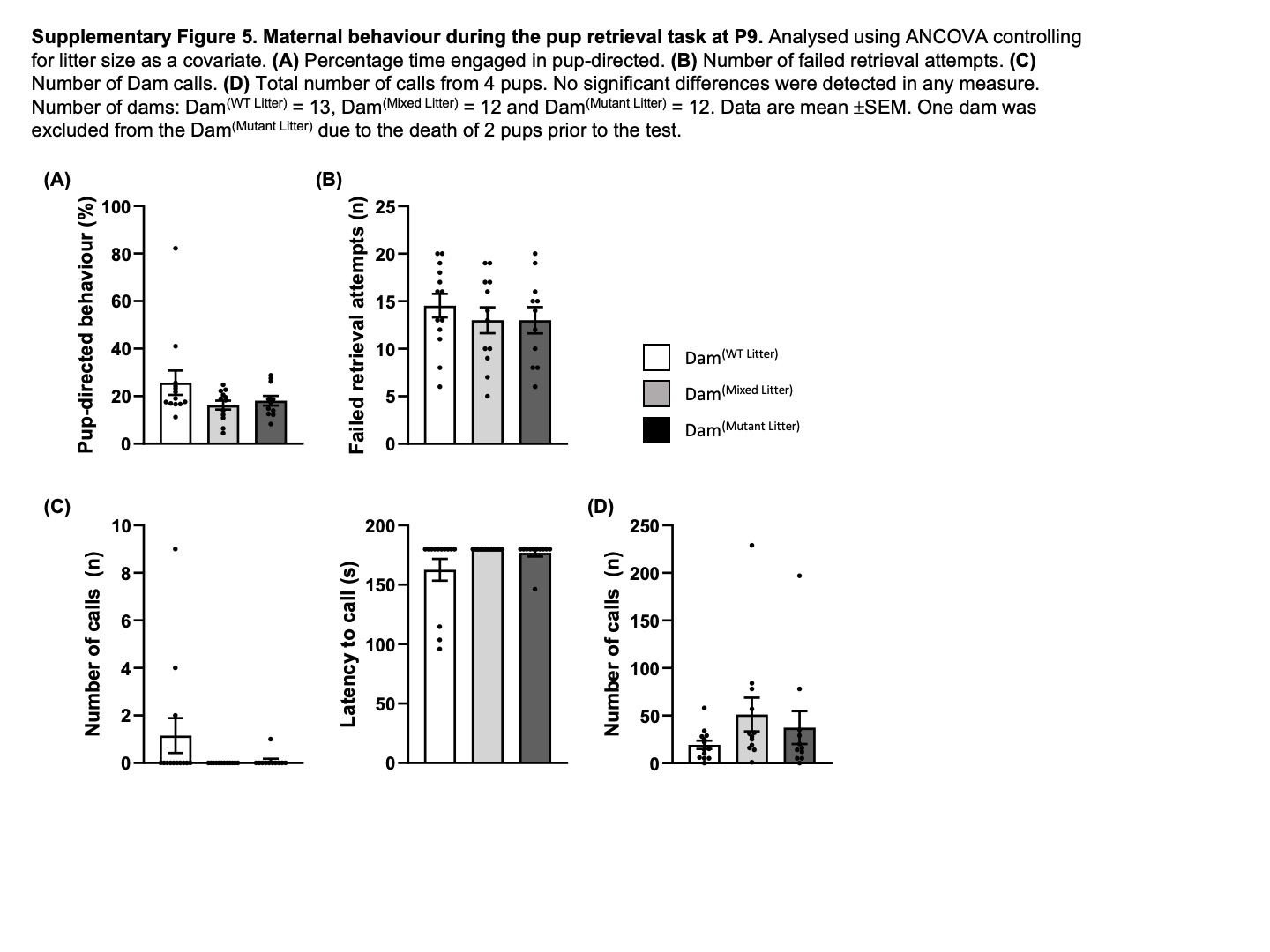

Supplement: Supplementary file 6 [file Image_5.JPEG]

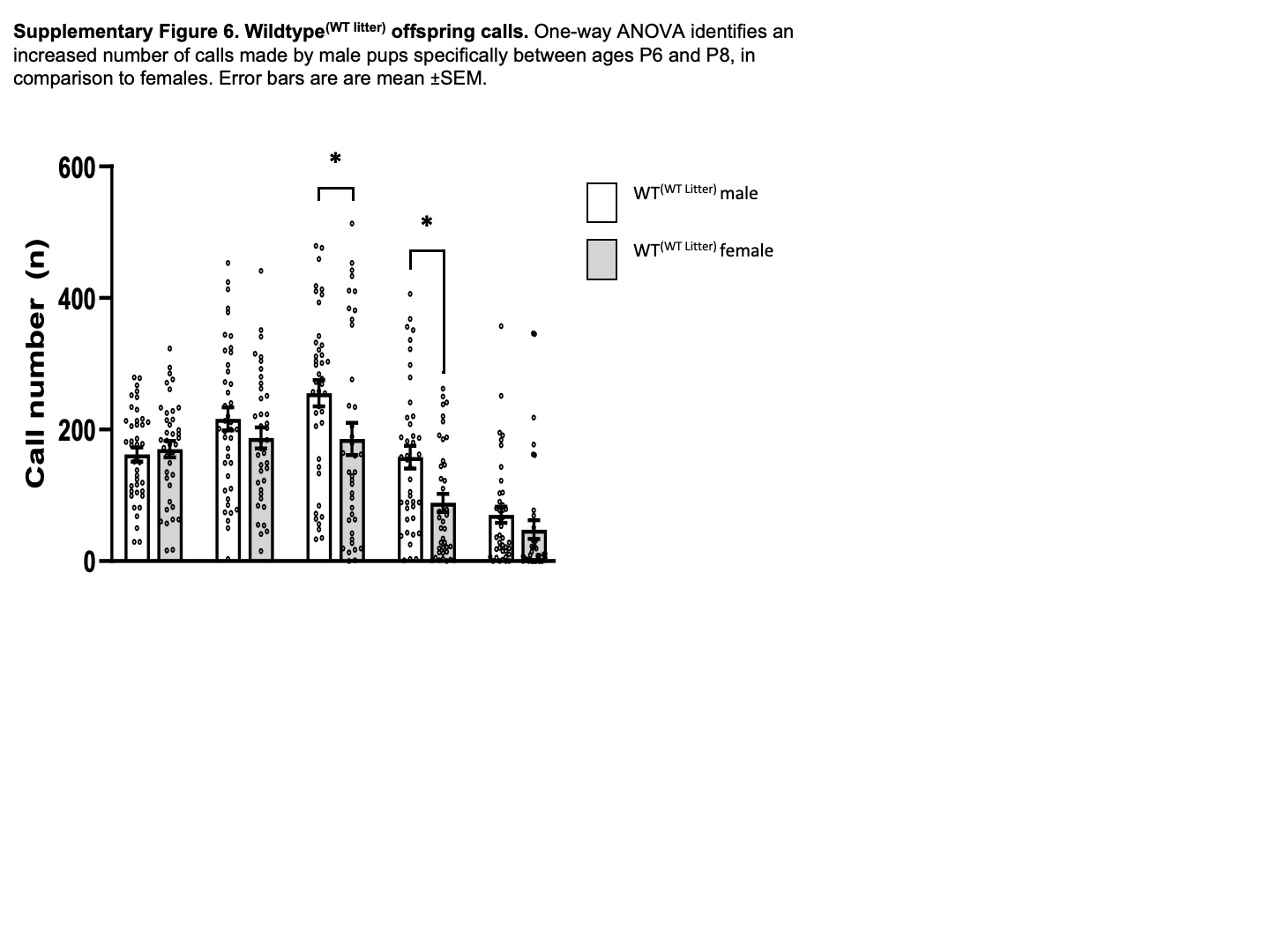

Supplement: Supplementary file 7 [file Image_6.JPEG]
